# Short-term exposure to ambient air pollution increased in-hospital non-ST-elevation myocardial infarction mortality risk, but not ST-elevation myocardial infarction: case-crossover based evidence from Beijing, China

**DOI:** 10.3389/fpubh.2025.1613082

**Published:** 2025-06-20

**Authors:** Yakun Zhao, Yuxiong Chen, Yanbo Liu, Siqi Tang, Yitao Han, Yuansong Zhuang, Jia Fu, Zhen’ge Chang, Xinlong Zhao, Jinyan Lei, Zhongjie Fan

**Affiliations:** ^1^Department of Cardiology, Peking Union Medical College Hospital, Peking Union Medical College, Chinese Academy of Medical Sciences, Beijing, China; ^2^Department of Internal Medicine, Peking Union Medical College Hospital, Peking Union Medical College, Chinese Academy of Medical Sciences, Beijing, China; ^3^Department of Healthcare, Peking Union Medical College Hospital, Peking Union Medical College, Chinese Academy of Medical Sciences, Beijing, China; ^4^Intensive Care Unit, The First Medical Center of Chinese PLA General Hospital, Beijing, China

**Keywords:** air pollution, acute myocardial infarction, ST-elevation myocardial infarction, non-ST-elevation myocardial infarction, in-hospital mortality, case-crossover

## Abstract

**Background:**

Previous studies have shown that air pollution affects the incidence of ST-segment elevation myocardial infarction (STEMI) and non-ST-segment elevation myocardial infarction (NSTEMI) differently. However, limited studies have examined the impact of air pollution on the mortality of these acute myocardial infarction (AMI) subtypes.

**Methods:**

Using AMI hospitalization data from Beijing (2013–2019), we applied a time-stratified case-crossover design with conditional Poisson regression models to evaluate associations between short-term exposure to six pollutants (PM_2.5_, PM_10_, SO_2_, NO_2_, CO, and O_3_) and daily in-hospital mortality for overall AMI, STEMI, and NSTEMI. Subgroup analyses based on demographics, comorbidities, and coronary artery disease (CAD) history were conducted to identify vulnerable populations. Additionally, a retrospective case–control analysis with multivariable logistic regression involved all AMI admission cases, was conducted to explore whether the association between air pollution exposure and in-hospital AMI mortality is independent of other mortality risk factors.

**Results:**

During the study period, there were 149,632 AMI admissions, with 10,983 in-hospital deaths (4,361 STEMI and 4,299 NSTEMI). Elevated levels of PM_2.5_, PM_10_, SO_2_, NO_2_, and CO on admission day were significantly associated with increased in-hospital mortality for overall AMI and NSTEMI, but not for STEMI. The effect of pollutants on NSTEMI mortality was greater in patients with old myocardial infarction (OMI) or percutaneous coronary intervention/coronary artery bypass grafting (PCI/CABG) history. In case–control analysis with multivariable logistic regression, increased pollutants concentration remained significantly associated with in-hospital NSTEMI mortality after adjusting for other mortality risk factors.

**Conclusion:**

Short-term exposure to PM_2.5_, PM_10_, SO_2_, NO_2_, and CO increases the risk of in-hospital AMI mortality, particularly for NSTEMI. Individuals with CAD history require more protective measures due to the vulnerability to air pollution.

## 1 Introduction

Cardiovascular diseases (CVD), including acute myocardial infarction (AMI), are the foremost cause of death and disability among the global population. The Global Burden of Disease (GBD) study in 2019 (1) reported that ischemic heart disease (IHD) resulted in 4.2 million deaths and 182 million disability-adjusted life years (DALYs) in 2019, comprising 7.19% of global DALYs. According to Annual Report on Cardiovascular Health and Diseases in China (2023), the AMI mortality rate among Chinese residents has been on the rise over the past decade. The AMI mortality rates for urban and rural residents increased from 47.36 per 100,000 and 48.53 per 100,000 in 2011 to 63.25 per 100,000 and 83.26 per 100,000 in 2021, respectively. Therefore, identifying risk factors for AMI mortality and implementing effective interventions to reduce the mortality risk in AMI patients is of great significance.

Numerous studies have indicated that higher air pollution levels, especially particulate matter, were associated with increased CVD mortality (2–5). Some research found that preadmission air pollution exposure is associated with higher in-hospital mortality risks (3, 6–8). However, previous research predominantly focuses on particulate matter’s impact on total CVD mortality or results of long-term exposure to air pollution (3–5, 9, 10), with limited attention to short-term effects of air pollution on AMI mortality, especially in-hospital mortality. A prior Beijing study on fine particulate matter and ischemic heart disease (IHD) (11) revealed that although out-of-hospital IHD deaths were 2–10 times more frequent than in-hospital deaths, fine particulate matter similarly elevated risks of out-of-and in-hospital mortality. Thus, we propose that exploring the association between short-term air pollution exposure and in-hospital AMI mortality provides a valid surrogate approach for assessing the effect of air pollution on overall AMI mortality.

As two different AMI subtypes, STEMI and NSTEMI exhibit distinct in pathogenesis and clinical features. Researchers have found that exposure to air pollutants increases the risk of STEMI rather than NSTEMI within 24 h (12), potentially due to air pollution triggers myocardial infarction through different mechanisms. Investigating whether this subtype-specific risk extends to mortality might clarify how air pollution contributes to AMI fatalities. However, current studies on the differences of air pollution’s impacts on STEMI and NSTEMI are limited. Most existing studies only focus on particulate matter and outcomes like emergency visits or hospital admissions (12–18), while few studies have compared the effect of air pollution on the mortality of AMI subtypes.

This study employed a case-crossover design to evaluate the short-term exposure to air pollution on daily in-hospital AMI mortality in Beijing, China. We analyzed overall and subtype-specific AMI mortality and identified susceptible populations through subgroup analysis.

## 2 Materials and methods

### 2.1 Background

Beijing (39° 56’ N, 116° 20′E), located in the northern China, has a temperate monsoon climate characterized by cold, dry winters and hot, humid summers. Similar to other northern Chinese cities, Beijing requires centralized heating during cold months. As the national capital and largest city, Beijing has approximately 21 million permanent residents. The electronic medical record system is widely used in Beijing, with standardized recording and reporting of front-page hospitalization records across all public and private hospitals. These rigorously quality-controlled records provide reliable clinical data for research.

### 2.2 Data collection

We obtained anonymized daily AMI (ICD-10, I21-I22) hospital admission records, covering all public and private hospitals in Beijing, between January 1st, 2013 and December 31st, 2019 from the Beijing Municipal Health Commission Information Center. The dataset comprised patient’s sex, age, address, occupation type, medical insurance type, marital status, admission date, length of stay, diagnoses, surgical/invasive procedures during hospitalization, blood transfusion, coma status, in-hospital death, etc. All data were de-identified to protect patient privacy. This study was approved by Peking Union Medical College Hospital (PUMCH) Institutional Review Board (approval number: S-K1097).

To account for potential variations in baseline environmental exposure, we selected permanent Beijing residents aged ≥20 years as the study population. In-hospital mortality was defined as in-hospital death occurring within ≤30 days after admission to assess the acute effects of air pollution. According to the patients’ diagnoses (coded by ICD-10) and in-hospital surgical/invasive procedures (coded by ICD-9-CM), we categorized AMI into STEMI (I21.0 – I21.3), NSTEMI (I21.4), and unspecified type, and collected information on comorbidities, previous cardiovascular diseases history, invasive therapies and surgery (see Supplementary Table 1 for complete ICD coding details).

Daily concentrations of PM_2.5_, PM_10_, SO_2_, NO_2_, CO, and O_3_ were collected from 12 national air quality monitoring stations [see Zhang et al. (19) for station location details] of China National Environmental Monitoring Centre, with the average of 24-h mean concentrations across these sites representing exposure. To account for potential confounders, we collected meteorological data (daily mean temperature, relative humidity, and air pressure) from the Beijing Meteorological Service’s station in the southern part of Beijing [see Zhang et al. (19) for station location detail] and influenza surveillance data from the Chinese National Influenza Center (see details in our previous study) (20). We also collected the dates of public holidays (e.g., the New Year’s Day, National Day) and heating period (usually from November 15th to March 15th) from public news.

### 2.3 Statistical analyses

As a widely used method for evaluating acute events (such as deaths), the case-crossover (CCO) design can effectively control time-invariant and time-stable confounders (such as age, sex, genetics, socioeconomic status) through self-matching. To assess the association between short-term exposure to air pollution preceding hospital admission and daily in-hospital AMI mortality in Beijing, this study employed a time-stratified case-crossover design, which can offer enhanced statistical efficiency and better control for temporal trends and seasonal influences by matching the case days to the same day of week in same year and month (21).

Since the in-hospital mortality of AMI is a rare event approximating a Poisson distribution, we employed a generalized linear model (GLM) with conditional Poisson regression to control for overdispersion and autocorrelation (22). We adjusted for meteorological factors (7-day averages of daily mean temperature, relative humidity, and air pressure), influenza epidemics, public holidays, and heating period were sequentially adjusted in the model. The final model was selected based on the minimum quasi-Poisson Akaike information criterion (Q-AIC) value and expressed as follows:


$$\log[E(Y_{t})]=\alpha +{\mathrm{\beta Z}}_{t}+\mathrm{ns}({\mathrm{MT}}_{t}\;\mathrm{lag}06,4)+\text{factor}(\text{holiday})+\text{factor}(\text{stratum})$$

In this formula, t represents day t of admission; E(Y_t_) is the number of in-hospital AMI death for patients admitted on day t; *α* is the intercept; β indicates the vector of coefficients for Z_t_; Z_t_ indicates the daily mean concentration of air pollutant on day t; MT_t_ lag06 are the 7-day averages of daily mean temperature preceding hospital admission (day t); ns(4) indicates a natural cubic spline function with 4 degrees of freedom being used to adjusted for non-linearity of daily mean temperature; factor (holiday) is a binary variable controlling for the confounding effect of public holidays; factor (stratum) represent the time-stratified term (same weekday, month, and year).

The lag effects of air pollutants on AMI in-hospital mortality were assessed by evaluating single-day lags (lagN, N = 0, 1, …, 5) and multi-day moving average lags (lag0N, N = 0, 1, …, 5). For each pollutant, the lag effect with greatest estimate was identified as the strongest effect of this pollutants.

Subgroup analyses were conducted to identify potentially susceptible populations and investigate potential modifying effects, stratified by sex, age (<65 years and ≥65 years), comorbidities [hypertension, diabetes, and chronic kidney disease (CKD)], and coronary artery disease (CAD) history [old myocardial infarction (OMI), and previous percutaneous coronary intervention (PCI)/coronary artery bypass grafting (CABG) history]. During the study period, Beijing’s air quality improved due to air pollution control policies taken by government. We stratified the analysis into two periods: 2013–2015 and 2016–2019, evaluating the impact of air pollution on health outcomes before and after governance interventions. Only the strongest effects in the single-day lag model were evaluated in subgroup analyses. The differences in effect estimates across subgroups were evaluated for significance by *Z*-test.

Sensitivity analyses were performed to assess the stability of the effect estimate. First, we built two-pollutant models adjusted for co-pollutants with a Spearman correlation coefficient *|r|* ≤ 0.7 to assess potential confounding effects of co-pollutants. Second, the degrees of freedom (dfs) for the natural cubic spline function of temperature were varied (3, 5, 6). Third, lag periods for daily mean temperature were altered to lag0, lag01, and lag03.

To validate whether the association between short-term exposure to air pollutants and in-hospital AMI mortality is independent of other mortality risk factors, we conducted a retrospective case–control analysis with multivariable logistic regression, which included all AMI admissions in Beijing from 2013 to 2019. This analysis used air pollutant concentrations preceding hospital admission as the independent variable and in-hospital mortality as the outcome variable, adjusting for demographic, clinical, and socioeconomic factors (details in Supplementary material).

In our primary CCO analysis, results present as relative risks (RRs) with 95% confidence intervals (CIs) per interquartile range (IQR) increase in pollutant concentrations. The multivariable logistic regression analysis presents odds ratio (ORs) with 95% CIs per IQR increase in pollutant. All statistical analyses were conducted using R (version 4.1.1) and “glm,” “splines,” etc. packages. *P*-value < 0.05 (two-sided) was defined as statistically significant.

## 3 Results

Among 149,632 AMI admissions in Beijing from 2013 to 2019, a total of 10,983 patients experienced in-hospital death within 30 days, including 4,361 STEMI, 4,299 NSTEMI and 2,323 unspecified AMI deaths. The 30-day in-hospital mortality rates for overall AMI, STEMI and NSTEMI admissions were 7.3, 6.2, and 6.2%, respectively. There was an average of 4.3 deaths per day for overall AMI, 1.71 for STEMI and 1.68 for NSTEMI. Over half of deceased patients were male (54.1%), and most were aged 65 years and older (87.6%) (Table 1). The most common comorbidity was hypertension (32.1%), followed by diabetes (20.1%). OMI was present in 13.9, and 8.3% had PCI/CABG history. Compared to patients died of STEMI, those died of NSTEMI were older (79.2 vs. 75.7 years), had longer hospital stays (8 vs. 5.9 days) and higher rates of OMI (19.5% vs. 10.2%) and PCI/CABG history (10.8% vs. 6.7%). More details on descriptive analysis of AMI patients are presented in Supplementary Table 2.

**Table 1:** Demographic and clinical characteristics of in-hospital AMI mortality.

Characteristics	Overall AMI	STEMI	NSTEMI	*p*-value^*^
	(*n* = 10983)	(*n* = 4361)	(*n* = 4299)	
Sex, *n* (%)				
Male	5942 (54.1)	2379 (54.6)	2299 (53.5)	0.316
Female	5041 (45.9)	1982 (45.4)	2000 (46.2)	
LOS (days), mean ± SD	7.0 ± 7.0	5.9 ± 6.6	8.0 ± 7.2	0.000
Age (years), mean ± SD	77.6 ± 10.7	75.7 ± 11.5	79.2 ± 9.6	0.000
Age, *n* (%)				
<65	1367 (12.4)	725 (16.6)	382 (8.9)	0.000
≥65	9616 (87.6)	3636 (83.4)	3917 (91.1)	
Comorbidities, *n* (%)				
Hypertension	3525 (32.1)	1482 (34.0)	1356 (31.5)	0.016
Diabetes	2203 (20.1)	874 (20.0)	875 (20.4)	0.717
CKD	1443 (13.1)	460 (10.6)	636 (14.8)	0.000
CAD history, *n* (%)				
OMI	1525 (13.9)	445 (10.2)	840 (19.5)	0.000
PCI/CABG history	908 (8.3)	294 (6.7)	465 (10.8)	0.000

*P-value for difference between STEMI and NSTEMI, *t*-test for comparison of continuous variables, chi-square test for categorical variables. *P*-values < 0.001 are shown as 0.000. LOS, length of stay; SD, standard deviation; CKD, chronic kidney disease; CAD, coronary artery disease; OMI, old myocardial infarction; PCI/CABG, percutaneous coronary intervention or coronary artery bypass grafting.

**Figure 1:**
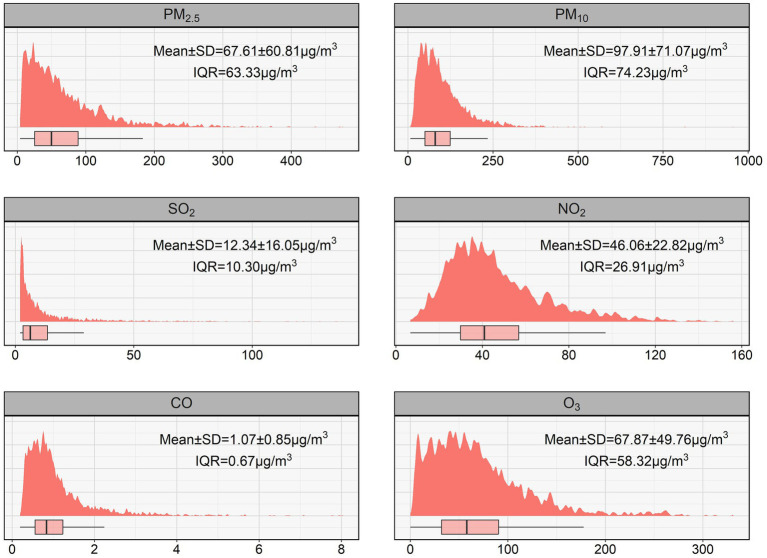
Summary of daily air pollution concentrations in Beijing during 2013–2019. IQR, interquartile range; SD, standard deviation.

During the period, the median (IQR) concentrations of PM_2.5_, PM_10_, SO_2_, NO_2_, CO, and O_3_ were 49.69(25.34–88.67), 80.5(49.92–124.15), 6.28(3.25–13.55), 40.92(29.88–56.79) μg/m^3^, 0.84(0.56–1.23) mg/m^3^, and 57.86(32.09–90.41) μg/m^3^, respectively (Figure 1). From 2013 to 2019, the concentrations of pollutants gradually decreased, except for ozone (Supplementary Figure 1). The mean (standard deviation, SD) of daily mean temperature, humidity, and air pressure during study were 13.78(11.23) °C, 51.45(19.87) %, and 1012.9(10.2) hPa, respectively (Supplementary Table 3). Spearman correlation analysis demonstrated strong correlations (*r* ≥ 0.7) among PM_2.5_, PM_10_, NO_2_, and CO, thus excluded from two-pollutant models due to strong collinearity. O_3_ exhibited a strong correlation with daily temperature (*r* = 0.72), while other pollutants showed weaker associations (|*r*| < 0.7) with meteorological factors (Supplementary Table 4).

**Figure 2:**
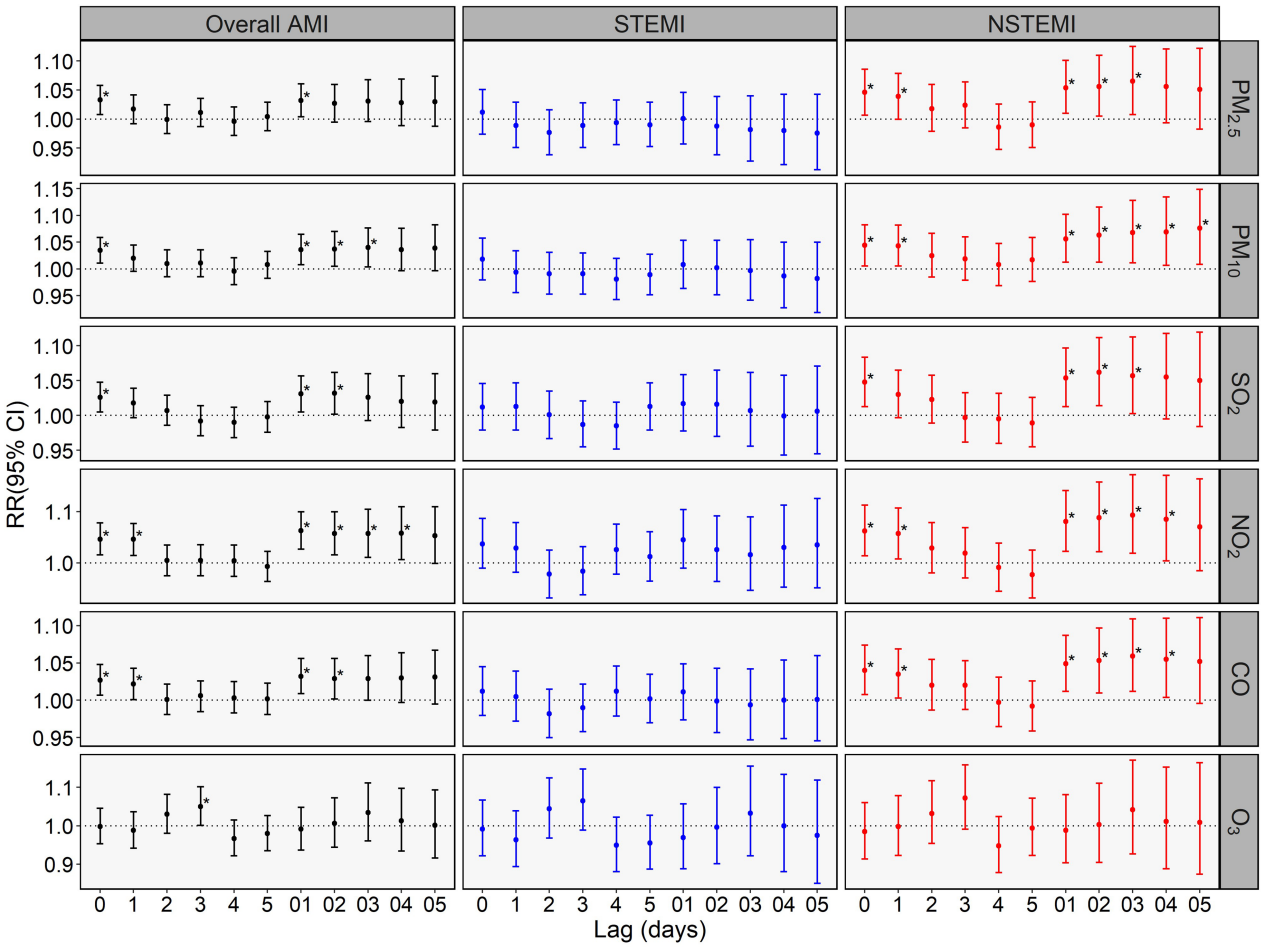
Relative risks (RRs) with 95% CIs of in-hospital mortality for overall AMI, STEMI, and NSTEMI per interquartile range (IQR) increase in air pollutant concentration in single pollutant model. *indicates *p* <0.05. The IQR of daily concentrations of PM_2.5_, PM_10_, SO_2_, NO_2_, CO, and O_3_ were 63.33 μg/m^3^, 74.23 μg/m^3^, 10.30 μg/m^3^, 26.91 μg/m^3^, 0.67 mg/m^3^ and 58.32 μg/m^3^, respectively.

Figure 2 presents the relative risks (RRs) of in-hospital mortality for overall AMI, STEMI, and NSTEMI per IQR increase in daily air pollutant concentrations in single-pollutant models, with detailed strongest effects in Table 2. Elevated levels of PM_2.5_, PM_10_, SO_2_, NO_2_, and CO were significantly associated with increased mortality for overall AMI and NSTEMI, but not for STEMI. In single-day lag models, the strongest effects of those five pollutants on overall AMI and NSTEMI occurred at lag0, then weaken gradually. In moving average lag models, these five pollutants showed longer-lasting effects, with delayed peak impacts on NSTEMI versus overall AMI mortality. Notably, PM_10_ showed the most prolonged effect on NSTEMI mortality, remaining significant with the strongest effect at lag05. Additionally, we found that elevated levels of O_3_ at lag3 were significantly associated with increased overall AMI mortality (RR = 1.050 per 58.32 μg/m^3^, 95% CI: 1.001–1.102), but no associations were observed for STEMI or NSTEMI. The lag days of the strongest effect for each pollutant were applied to subsequent subgroup analyses and two-pollutant models.

**Table 2:** The strongest effects of air pollutants on in-hospital mortality for overall AMI, STEMI, and NSTEMI in single-and multi-day lag models.

Pollutants	Overall AMI		STEMI		NSTEMI	
PM_2.5_	lag0	1.033 (1.008,1.058)*	lag0	1.012 (0.974,1.051)	lag0	1.046 (1.007,1.086)*
	lag01	1.032 (1.004,1.061)*	lag01	1.001 (0.957,1.046)	lag03	1.065 (1.008,1.125)*
PM_10_	lag0	1.035 (1.011,1.059)*	lag0	1.018 (0.980,1.058)	lag0	1.044 (1.006,1.083)*
	lag03	1.040 (1.004,1.077)*	lag01	1.008 (0.964,1.054)	lag05	1.076 (1.009,1.149)*
SO_2_	lag0	1.026 (1.005,1.048)*	lag5	1.013 (0.979,1.047)	lag0	1.048 (1.013,1.084)*
	lag02	1.032 (1.002,1.062)*	lag01	1.017 (0.978,1.059)	lag02	1.062 (1.014,1.112)*
NO_2_	lag0	1.046 (1.016,1.078)*	lag0	1.037 (0.990,1.087)	lag0	1.062 (1.014,1.113)*
	lag01	1.063 (1.027,1.100)*	lag01	1.045 (0.990,1.104)	lag03	1.093 (1.019,1.172)*
CO	lag0	1.027 (1.007,1.048)*	lag0	1.012 (0.980,1.045)	lag0	1.040 (1.008,1.074)*
	lag01	1.032 (1.009,1.056)*	lag01	1.011 (0.974,1.049)	lag03	1.059 (1.012,1.109)*
O_3_	lag3	1.050 (1.001,1.102)*	lag3	1.065 (0.989,1.148)	lag3	1.072 (0.991,1.159)
	lag03	1.034 (0.961,1.112)	lag03	1.033 (0.922,1.156)	lag03	1.042 (0.927,1.171)

* indicates *p* < 0.05. The effect estimates present as the relative risks (RRs) with 95% confidential intervals (CIs) of hospital admission for total AMI, STEMI, and NSTEMI per interquartile range (IQR) increase in pollutant concentration. The IQR of daily concentrations of PM_2.5_, PM_10_, SO_2_, NO_2_, CO, and O_3_ were 63.33 μg/m^3^, 74.23 μg/m^3^, 10.30 μg/m^3^, 26.91 μg/m^3^, 0.67 mg/m^3^ and 58.32 μg/m^3^, respectively.

**Figure 3:**
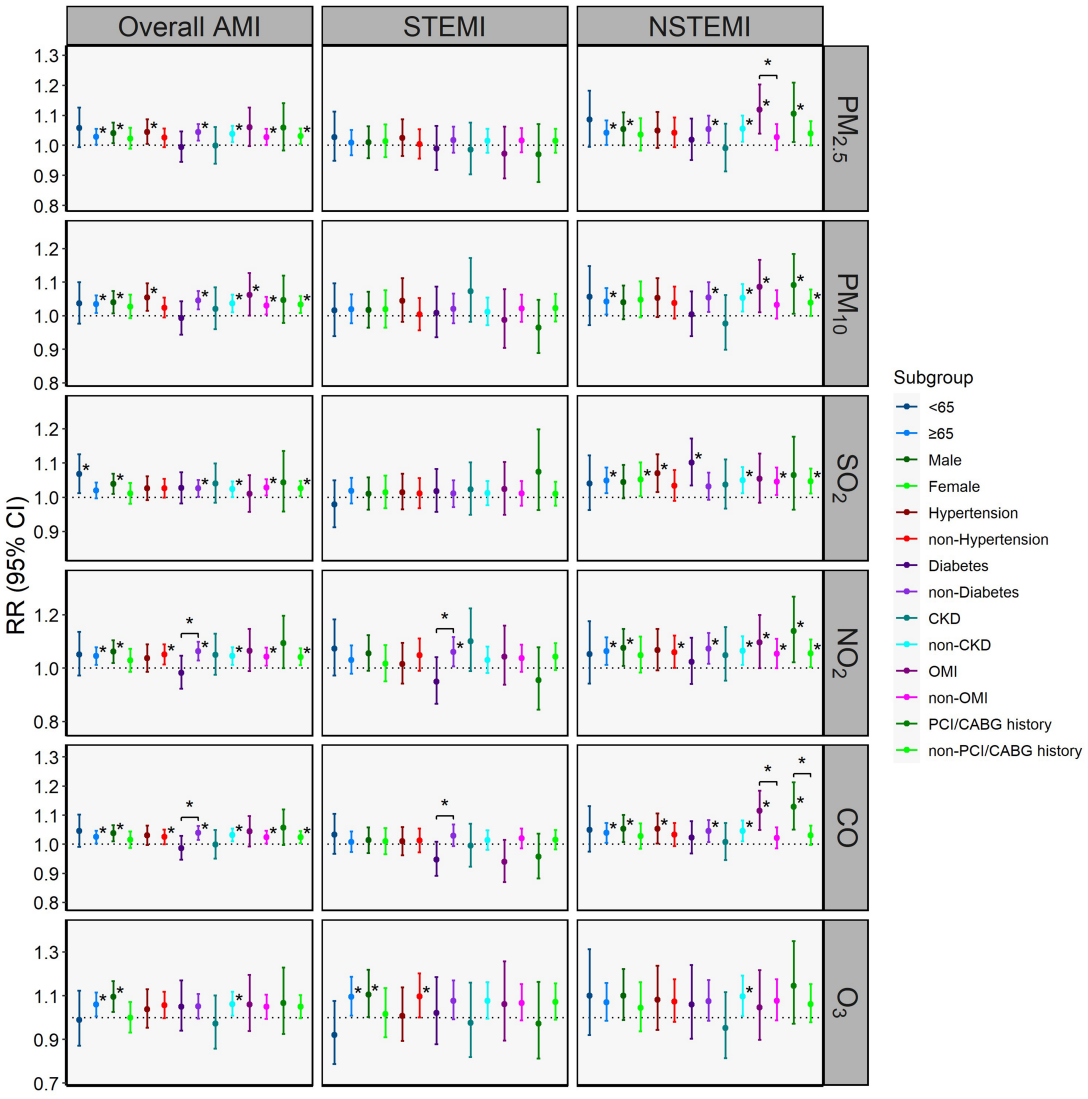
Relative risks (RRs) with 95% CIs of in-hospital mortality for overall AMI, STEMI, and NSTEMI per interquartile range (IQR) increase in air pollutant concentration: subgroup analyses. CKD, chronic kidney disease; OMI, old myocardial infarction; PCI/CABG, percutaneous coronary intervention or coronary artery bypass grafting. *indicates *p* <0.05. Brackets and asterisk (*) indicate statistical significance of Z tests for between-group comparisons (P_z_ <0.05). The IQR of daily concentrations of PM_2.5_, PM_10_, SO_2_, NO_2_, CO, and O_3_ were 63.33 μg/m^3^, 74.23 μg/m^3^, 10.30 μg/m^3^, 26.91 μg/m^3^, 0.67 mg/m^3^ and 58.32 μg/m^3^, respectively.

The results of subgroup analyses stratified by demographic characteristics and comorbidities are presented in Figure 3 and Supplementary Tables 5–11. We found that elevated O_3_ levels at lag3 were significantly associated with increased in-hospital STEMI mortality in older adults (≥65 years) (RR = 1.095 per 58.32 μg/m^3^, 95% CI: 1.010–1.187) and males (RR = 1.105 per 58.32 μg/m^3^, 95% CI: 1.002–1.219). However, no significant differences in the effects of air pollutants on in-hospital mortality for all AMI types were observed between sexes or age groups. For comorbidities, elevated NO_2_ and CO levels showed a positive correlation with increased mortality risks for overall AMI and STEMI in non-diabetic individuals, while displaying a negative correlation in diabetic individuals, showing a distinctly opposite trend (*P*_z_ < 0.05). No significant between-group differences were found in either hypertensive or CKD subgroups (*P*_z_ > 0.05). Compared to individuals without a history of OMI or PCI/CABG, elevated levels of PM_2.5_, PM_10_, SO_2_, NO_2_, and CO showed stronger associations with increased on overall AMI and NSTEMI mortality risk in those with prior OMI or PCI/CABG, while the between-group difference reached statistical significance for PM_2.5_ and CO on NSTEMI (*P*_z_ < 0.05). Subgroup analyses stratified by periods (Supplementary Figure 2) showed the effects of PM_2.5_, PM_10_, SO_2_, NO_2_, and CO on in-hospital mortality for NSTEMI were slightly decreased and became non-significant from 2013–2015 to 2016–2019, while no significance was found between different periods (*P*_z_ > 0.05).

**Figure 4:**
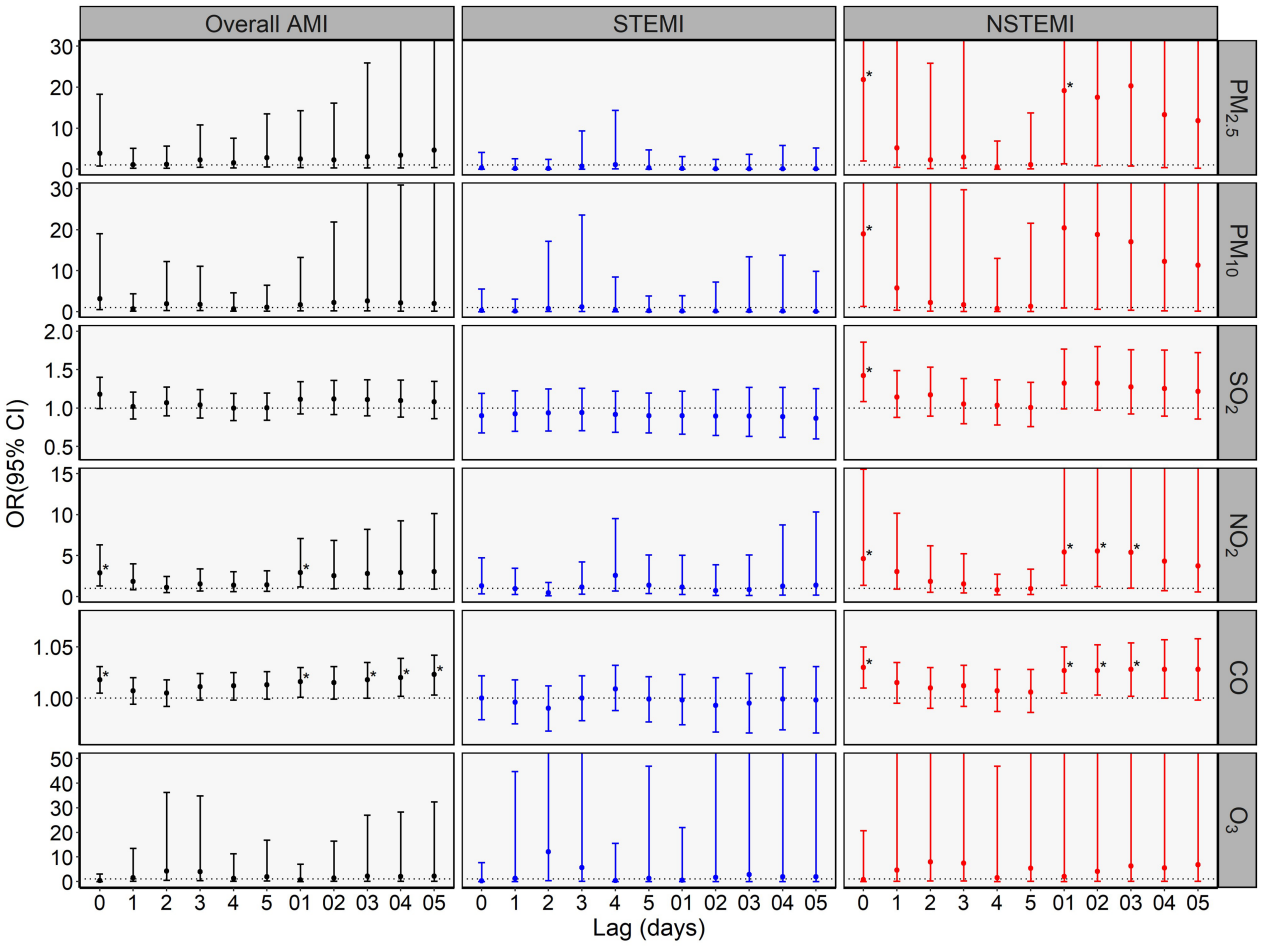
Odds ratios (ORs) with 95% CIs of in-hospital mortality among overall AMI, STEMI, and NSTEMI per interquartile range (IQR) increase in air pollutant concentration in multivariable logistic regression in case–control analysis. * indicates p <0.05. The IQR of daily concentrations of PM_2.5_, PM_10_, SO_2_, NO_2_, CO, and O_3_ were 63.33μg/m3, 74.23μg/m3, 10.30μg/m3, 26.91μg/m3, 0.67mg/m3 and 58.32μg/m3, respectively.

In two-pollutant models (Table 3), after adjusting for co-pollutants (excluding O_3_), the effect of PM_2.5_, PM_10_, NO_2_, SO_2_, and CO were decreased and no longer significant, except for the effect of NO_2_ in moving average lag models which retained significance. For O_3_, the association with increased overall AMI mortality at lag3 is no longer significant after adjusting for PM_2.5_ and SO_2_. In the remaining sensitivity analyses (Supplementary Figures 3, 4), our results remained robust except for the association between O₃ at lag3 and AMI mortality, which became non-significant after temperature lag period adjustment (Figure 4).

**Table 3:** Relative risks with 95% CIs of in-hospital mortality for overall AMI per interquartile range (IQR) increase in air pollutant concentration in two-pollutant model.

Pollutants	Model	Single-day lag^a^	Multi-day lag^b^
PM_2.5_	Unadjusted	1.033 (1.008,1.058)*	1.032 (1.004,1.061)*
	+SO_2_	1.023 (0.994,1.054)	1.022 (0.991,1.054)
	+O_3_	1.034 (1.010,1.060)*	1.033 (1.005,1.062)*
PM_10_	Unadjusted	1.035 (1.011,1.059)*	1.040 (1.004,1.077)*
	+SO_2_	1.027 (0.999,1.055)	1.032 (0.996,1.069)
	+O_3_	1.036 (1.011,1.06)*	1.039 (1.004,1.076)*
SO_2_	Unadjusted	1.026 (1.005,1.048)*	1.032 (1.002,1.062)*
	+PM_2.5_	1.014 (0.989,1.041)	1.017 (0.983,1.051)
	+PM_10_	1.014 (0.989,1.039)	1.016 (0.984,1.050)
	+NO_2_	1.008 (0.980,1.037)	1.014 (0.982,1.048)
	+CO	1.013 (0.987,1.041)	1.017 (0.984,1.051)
	+O_3_	1.028 (1.006,1.051)*	1.034 (1.004,1.064)*
NO_2_	Unadjusted	1.046 (1.016,1.078)*	1.063 (1.027,1.100)*
	+SO_2_	1.039 (0.999,1.080)	1.057 (1.014,1.101)*
	+O_3_	1.051 (1.019,1.083)*	1.066 (1.029,1.104)*
CO	Unadjusted	1.027 (1.007,1.048)*	1.032 (1.009,1.056)*
	+SO_2_	1.019 (0.994,1.046)	1.024 (0.998,1.052)
	+O_3_	1.029 (1.008,1.050)*	1.033 (1.009,1.058)*
O_3_	Unadjusted	1.050 (1.001,1.102)*	1.034 (0.961,1.112)
	+PM_2.5_	1.049 (1.000,1.101)	1.029 (0.957,1.106)
	+PM_10_	1.052 (1.003,1.104)*	1.033 (0.961,1.110)
	+SO_2_	1.050 (1.000,1.101)	1.034 (0.962,1.112)
	+NO_2_	1.052 (1.002,1.104)*	1.047 (0.974,1.126)
	+CO	1.050 (1.001,1.102)*	1.040 (0.968,1.119)

* indicates *p* < 0.05. The lag times of pollutants were selected based on the strongest lagged effects in the single-pollutant model: ^a^single-day lag model: the lag days of PM_2.5_, PM_10_, SO_2_, NO_2_, and CO were selected as lag0, and the lag days of O_3_ were selected as lag3. ^b^Moving average lag model: the lag days of PM_2.5_, NO_2_, and CO were selected as lag01, the lag days of SO_2_ were selected as lag02, and the lag days of PM_10_ and O_3_ were selected as lag03. The IQR of daily concentrations of PM_2.5_, PM_10_, SO_2_, NO_2_, CO, and O_3_ were 63.33 μg/m^3^, 74.23 μg/m^3^, 10.30 μg/m^3^, 26.91 μg/m^3^, 0.67 mg/m^3^ and 58.32 μg/m^3^, respectively.

In retrospective case–control analysis with multivariable logistic regression (Supplementary Table 12), after adjusting potential risk factors of mortality, only NO_2_ and CO were significantly associated with in-hospital mortality among overall AMI patients, while PM_2.5_, PM_10_, SO_2_, NO_2_, and CO remained significantly associated with in-hospital mortality among NSTEMI patients. No significant association was found between elevated pollutant concentrations and in-hospital mortality among STEMI patients.

## 4 Discussion

In this 7-year retrospective study, we studied the association of short-term exposure to air pollutants with in-hospital mortality for overall AMI, STEMI and NSTEMI. We found that elevated levels of PM_2.5_, PM_10_, SO_2_, NO_2_ and CO were significantly associated with increased in-hospital mortality for overall AMI and NSTEMI, but not with that for STEMI. Moreover, these pollutants increased risk of NSTEMI in-hospital mortality independently of sex, age, comorbidities, complications, in-hospital treatment, and socioeconomic status. Specific subgroups, such as individuals with prior OMI or coronary revascularization history (PCI/CABG), exhibit increased susceptibility to the adverse effects of air pollutants.

Several studies showed that short-term exposure to particulate matter and gaseous pollutants with increased mortality risk of AMI or total CVDs (10, 17), but few have explored pollutants’ impact on AMI in-hospital mortality. An Iranian study (23) reported that elevated concentrations of SO_2_, CO, and O_3_ were significantly associated with increased in-hospital mortality risk for AMI patients, and these associations were independent of sex, age, and underlying health conditions. Studies based on hospitalization data from four Chinese provinces (Shanxi, Sichuan, Guangxi, and Guangdong) found that short-term exposure to particulate matter of different sizes (24) and PM_2.5_ chemical components (25) was related with higher risks of in-hospital mortality in AMI patients. A Portland study demonstrated that acute coronary syndrome patients undergoing coronary angioplasty had a higher risk of 30-day all-cause mortality when exposed to elevated PM_10_ and SO_2_ levels before admission (26). Conversely, there was no significant association between weather, air pollution and in-hospital mortality of MI patients in a study from São Paulo, Brazil (27). In our study, not only particulate matter but also gaseous pollutants exposures were significantly associated with increased in-hospital AMI mortality. Current evidence indicates that smaller particulate matter exerts stronger cardiovascular effects (24, 28). When our results presented as RR for an increase of 10 μg/m^3^ in pollutant’s concentrations, PM_2.5_ showed slightly greater estimates of in-hospital mortality than PM_10_ for NSTEMI (RR = 1.007, 95%CI: 1.001–1.013 vs. RR = 1.006, 95%CI: 1.001–1.011). Compared with other studies, the in-hospital mortality rate of AMI patients in our study was higher (7.3% vs. 2.2–5.6%), which may be attributed to the shorter time from symptom onset to admission due to the green channel policy in Beijing, and the preference of patients in Beijing to pass away in the hospital. Differences in study populations and environmental data might contribute to the observed variations in results across different studies.

As two subtypes of AMI, STEMI and NSTEMI differ in their pathogenesis and mortality risks. However, few studies have compared the effects of air pollution on mortality risks of these two AMI subtypes. Our study found that higher concentrations of PM_2.5_, PM_10_, SO_2_, NO_2_, and CO were significantly associated with increased in-hospital mortality risks for NSTEMI but not STEMI. Similarly, a case-crossover study based on Myocardial Ischemia National Audit Project (MINAP) database reported that elevated NO_2_ levels were associated with increased in-hospital mortality risks for NSTEMI and overall mortality, but not for STEMI (17). However, short-term exposure to particulate matter was significantly associated with increased in-hospital mortality risks for both STEMI and NSTEMI in the study based on AMI hospitalization data from four Chinese provinces (24). A study from Spain found that short-term exposure to PM_2.5_ and PM_10_ was associated with higher 24-h mortality risks following hospital admission for STEMI patients (29). In the study from Taiwan, China, elevated levels of PM_10_ in cold season and NO_2_ in warm season were associated with increased risk of in-hospital mortality risks in STEMI patients (30).

Compared to NSTEMI, STEMI is usually rapid-onset and severe, with higher in-hospital mortality risk (23). Consistently, our study observed shorter admission-to-death intervals among STEMI patients. We hypothesize that the differential effects of air pollution on STEMI and NSTEMI mortality risks may be attributed to the following reasons. First, the mortality risk in STEMI patients is primarily driven by disease (23), potentially overshadowing weaker influence of air pollution. Second, air pollutants may increase AMI mortality risk through mechanisms such as oxidative stress, systemic inflammatory response, vascular dysfunction, and enhanced thrombogenesis (10, 31). Mechanisms such as systemic inflammation and oxidative stress may require time to manifest their effects. As a result, the impact of air pollutants on NSTEMI in-hospital mortality is more pronounced, while effects on STEMI are only detectable in specific populations and under certain timeframes.

Previous studies have consistently shown that individuals with certain comorbidities (e.g., hypertension, COPD) or cardiovascular disease history have significantly increased risks of AMI and other CVD mortality when exposed to air pollutants (7, 32–34). Individuals with existing CAD were more likely to develop AMI when exposed to air pollutant like particulate matter (35, 36). Similarly, our study demonstrated more pronounced effects of air pollution on in-hospital NSTEMI mortality in patients with prior CAD (OMI or PCI/CABG) history. What’s more, we found that elevated NO₂ and CO concentrations were associated with higher in-hospital mortality for overall AMI and STEMI in non-diabetic individuals, whereas inverse associations were observed in diabetic individuals. This inverse association might be explained by the cardioprotective, antioxidant, and anti-inflammatory properties of antidiabetic medications (37–39), which could potentially attenuate the adverse impact of air pollution on AMI mortality in diabetic individuals. Further research was needed to explore how baseline health status and medications may modify individual susceptibility to air pollution-related health effects. During the study period, the concentrations of pollutants (except for O_3_) gradually decreased, and their associations with in-hospital NSTEMI mortality showed a weakening trend, though this change was not statistically significant. Future studies should evaluate the effect of air pollution control measures on pollution related mortality.

This study has several strengths. First, it is one of the few studies to analyze the impact of short-term exposure to air pollutants on in-hospital mortality for AMI and different AMI subtypes (STEMI and NSTEMI). It also explores the influence of comorbidities and CAD history on patient susceptibility. The study utilized 7-year hospitalization data from the entire Beijing area, minimizing selection bias and enhancing the stability and generalizability of the findings. Second, the time-stratified case-crossover design was employed to mitigate the effects of long-term and seasonal trends, while controlling for time-invariant confounders. Furthermore, a retrospective case–control analysis using multivariable logistic regression analyses, adjusted for potential mortality confounders, was using to validate whether the association between short-term exposure to air pollutants and in-hospital AMI mortality is independent of other mortality risk factors.

This study also has several limitations. First, using fixed-site exposure measurements as a proxy for individual exposure and considering preadmission exposure as the total exposure may lead to exposure bias. Second, the retrospective analysis based on admission records and the use of a daily time scale may overlook more acute effects occurring within hours. Third, this study did not consider the potential confounding effects of indoor air pollution sources (such as second-hand-smoke) or Beijing’s smoke-free law in 2015, which requires a 100% ban on indoor smoking in medical institutions and other public places. Additionally, the study focused solely on in-hospital AMI mortality, excluding prehospital and post-discharge deaths. Thus, our results might reflect the correlation between short-term air pollution exposure and AMI mortality, but cannot precisely estimate the total disease burden from air pollutant-related AMI mortality. Finally, the OR values for some pollutants derived from case–control study were higher than the RR values from the main CCO study. This discrepancy can be attributed not only to the distinct study designs and populations (the ecological CCO study assessed overall population risk, while the case–control study focused on individuals already hospitalized), but also to the large variability in pollutant concentrations. When we treated pollutant concentrations as continuous variables in case–control analysis with logistic regression, it led to wider confidence intervals and higher OR values. Future studies employing similar designs should be improved.

## 5 Conclusion

Short-term exposure to PM_2.5_, PM_10_, SO_2_, NO_2_, and CO increases the risk of in-hospital AMI mortality, particularly for NSTEMI rather than STEMI. Stratified analyses by AMI subtype (STEMI/NSTEMI) are recommended when assessing air pollution’s impact on AMI mortality. Patients with a history of CAD are more vulnerable to the adverse effects of pollutants on AMI mortality and require more protective measures.

## Data Availability

The original contributions presented in the study are included in the article/Supplementary material, further inquiries can be directed to the corresponding author.
