# Supplementary material for: Short-term exposure to ambient air pollution increased in-hospital non-ST-elevation myocardial infarction mortality risk, but not ST-elevation myocardial infarction: case-crossover based evidence from Beijing, China
Source: Front Public Health. 2025 Jun 20;13:1613082. doi: 10.3389/fpubh.2025.1613082 (PMC12226560; doi:10.3389/fpubh.2025.1613082)
Supplement: Supplementary file 1 [file Table_1.docx]

Supplementary Material

# Multivariable logistic regression in retrospective case-control analysis

In the multivariable logistic regression in retrospective cross-sectional analysis, we involved all acute myocardial infarction (AMI) admissions between 2013-2019 in Beijing to assess the association between increased concentrations of six air pollutants preceding admission and 30-day in-hospital mortality among AMI patients. This analysis used air pollutant concentrations preceding hospital admission as the independent variable. The outcome defined as in-hospital mortality with 30 days after admission. Cases were defined as patients with AMI who died within 30 days of hospital admission, while controls were defined as patients with AMI who survived beyond 30 days or were discharged alive. No matching was performed between cases and controls. Potential risk factors for death, such as demographic, clinical, and socioeconomic factors, were adjusted as covariates. The model structure is as follows:

$\boldsymbol{Logit}\left( \boldsymbol{Y} \right)\boldsymbol{= \alpha+}\boldsymbol{\beta}_{\boldsymbol{0}}\boldsymbol{X}_{\boldsymbol{0}}\boldsymbol{+}\boldsymbol{\beta}_{\boldsymbol{1}}\boldsymbol{X}_{\boldsymbol{1}}\boldsymbol{+}\boldsymbol{\beta}_{\boldsymbol{2}}\boldsymbol{X}_{\boldsymbol{2}}\boldsymbol{+\ldots}\boldsymbol{+}\boldsymbol{\beta}_{\boldsymbol{i}}\boldsymbol{X}_{\boldsymbol{i}}$ **(S1)**

In this formula , Y is the dependent variable, a binary variable for in-hospital mortality within 30 days after admission; X_0_ is the independent variable, a continuous variable of a pollutant's daily mean concentration, standardized by dividing by its interquartile range (IQR); β_0_ is regression coefficients of X_0_, the estimate of an IQR increase in the pollutant on risk of 30-day in-hospital mortality; X_1_, X_2_, ……, X_i_ are the potential covariables, including:

a. Meteorological factor: the 7-day averages of daily mean temperature on admission day and previous 6 days;

b. Demographic characteristics: sex (binary variable) and age (continuous variable);

c. Risk factors of cardiovascular disease (binary): hypertension, diabetes, dyslipidemia, chronic kidney disease (CKD);

d. Cardiovascular diseases (binary): old myocardial infarction (OMI), percutaneous coronary intervention history/coronary artery bypass grafting (PCI/CABG) history, atrial flutter/atrial fibrillation, stroke;

e. Chronic respiratory diseases (binary): chronic obstructive pulmonary disease (COPD), asthma;

f. Complications and other comorbidities (binary): heart failure, AMI mechanical complications, ventricular tachycardia/ventricular fibrillation, pulmonary infection, coma (binary);

g. In-hospital treatments: ventilation use (binary), blood transfusion (binary), intra-aortic balloon pump (IABP) use (binary), revascularization treatment (categorical variable);

h. Socioeconomic status (categorical): medical insurance type, marital status, occupation.

# Supplementary Figures and Tables

## Supplementary Figures
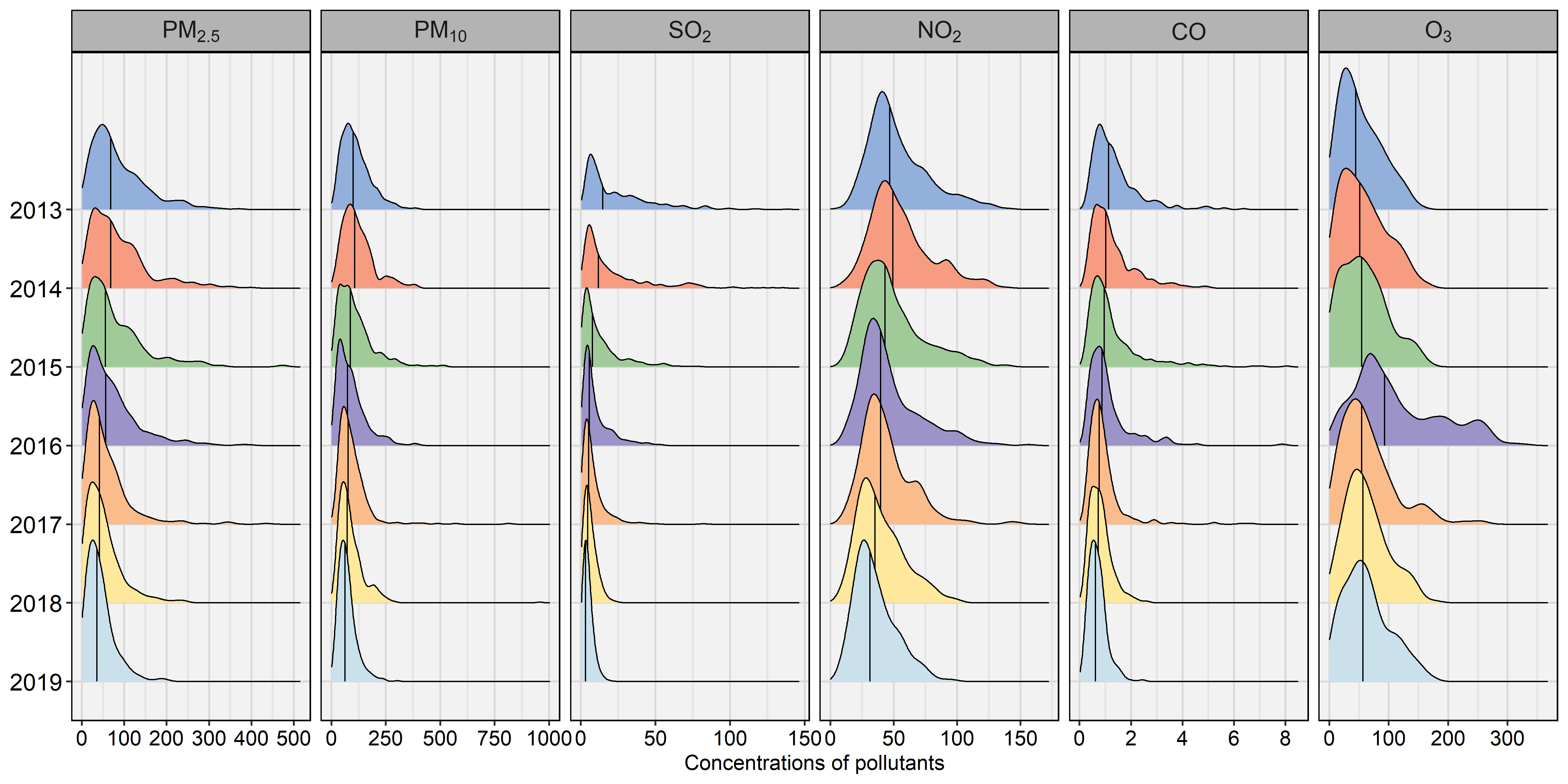


**Supplementary Figure 1.** Summary of daily air pollution concentrations in Beijing from 2013 to 2019 by year. Note: The black vertical lines represent the medians of pollutants concentrations.


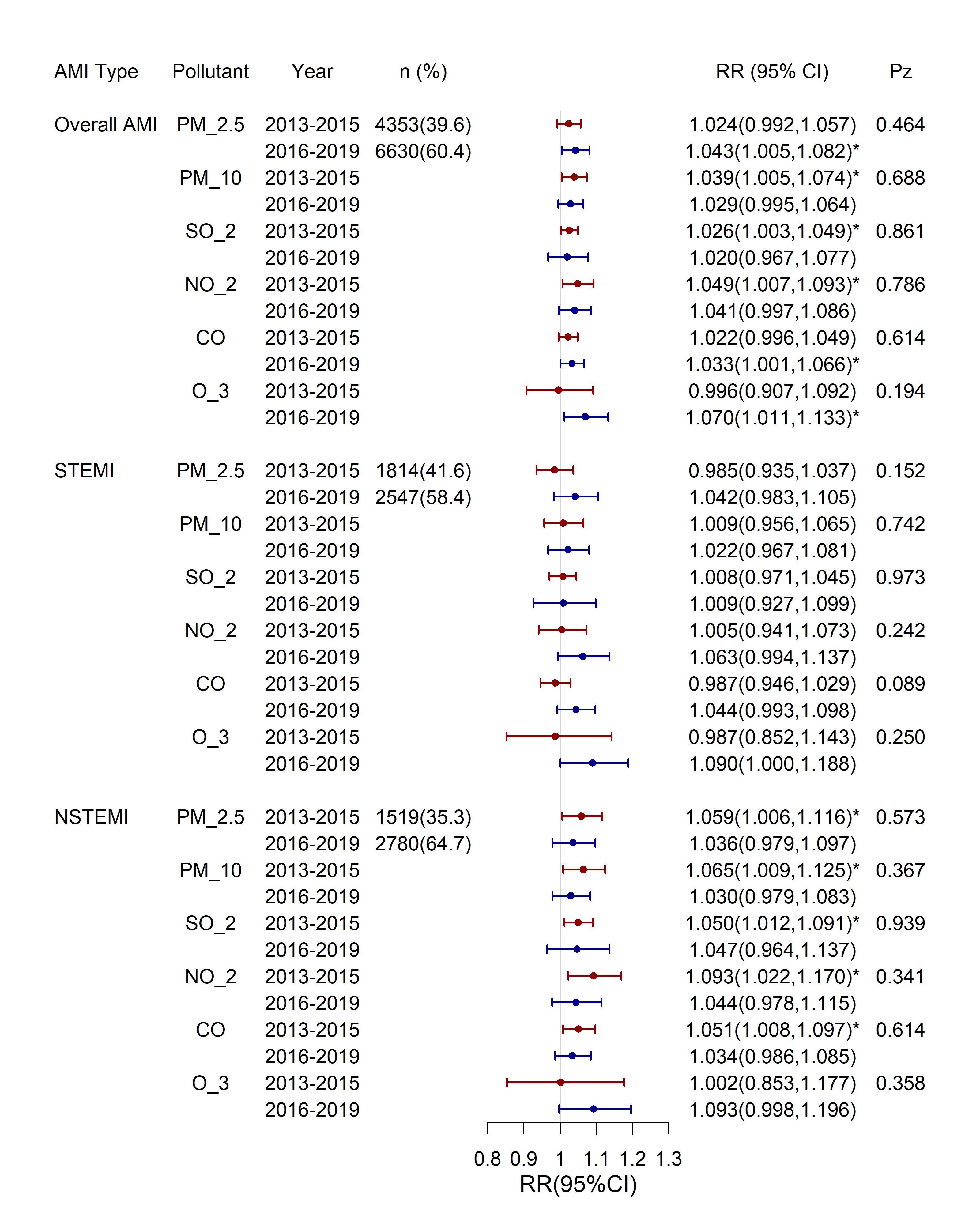


**Supplementary Figure 2.** Relative risks (RRs) with 95% CIs of in-hospital mortality for overall AMI, STEMI, and NSTEMI per interquartile range (IQR) increase in air pollutant concentration: stratified by periods. Note: *P_z_* indicates *P* value for the between-group comparisons by Z test. The IQR of daily concentrations of PM_2.5_, PM_10_, SO_2_, NO_2_, CO, and O_3_ were 63.33μg/m^3^, 74.23μg/m^3^, 10.30μg/m^3^, 26.91μg/m^3^, 0.67mg/m^3^ and 58.32μg/m^3^, respectively. Based on the strongest effects in the single-day lag model, the lag days of PM_2.5_, PM_10_, NO_2_, and CO were selected as lag0. The lag day of O_3_ was selected as lag3.


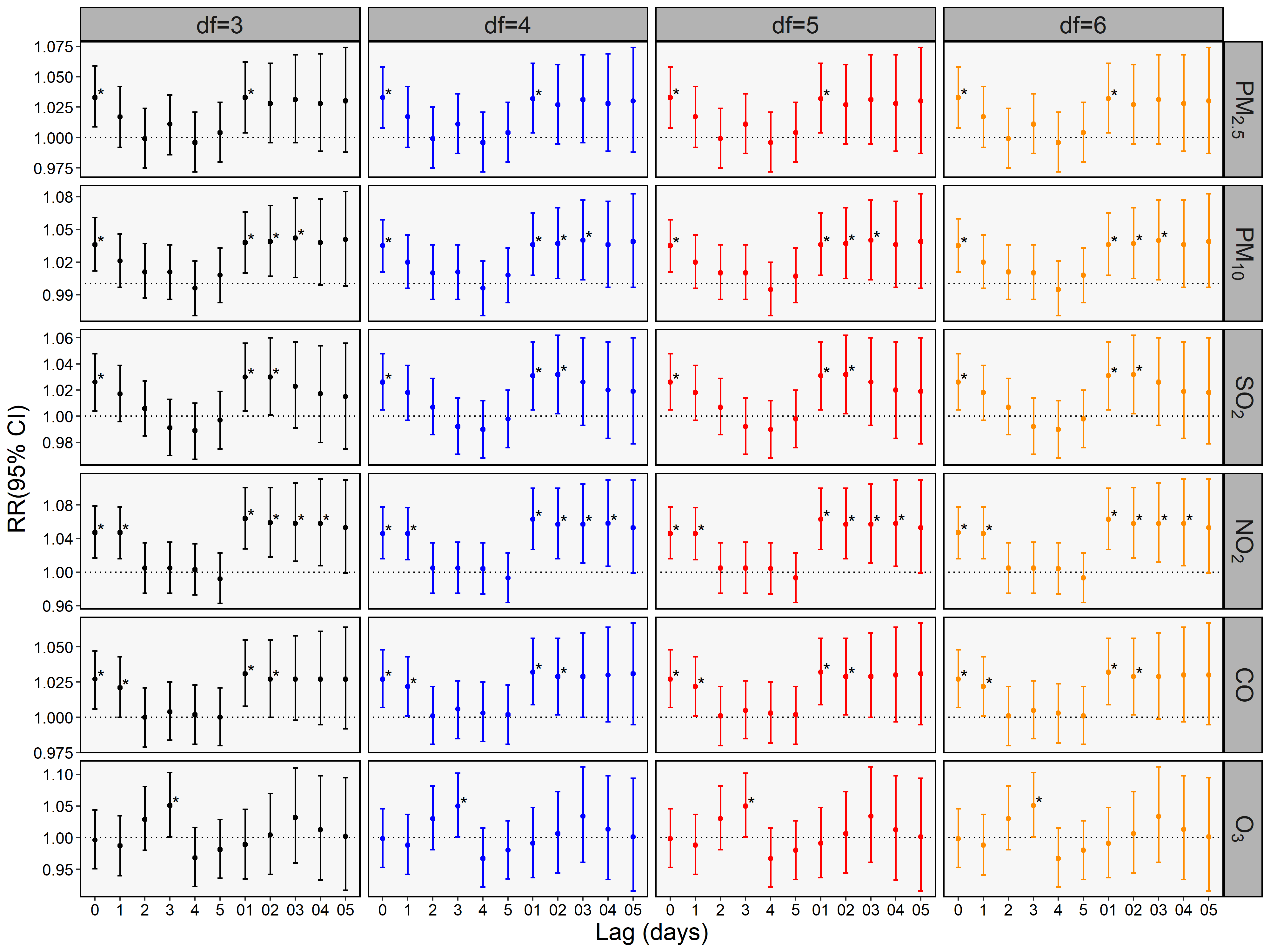


**Supplementary Figure 3.** Relative risks (RRs) with 95% CIs of in-hospital mortality for overall AMI per interquartile range (IQR) increase in air pollutant concentration adjusted degrees of freedom (dfs) of spline function for temperature. Note: * indicates *P* <0.05. The IQR of daily concentrations of PM_2.5_, PM_10_, SO_2_, NO_2_, CO, and O_3_ were 63.33μg/m^3^, 74.23μg/m^3^, 10.30μg/m^3^, 26.91μg/m^3^, 0.67mg/m^3^ and 58.32μg/m^3^, respectively.


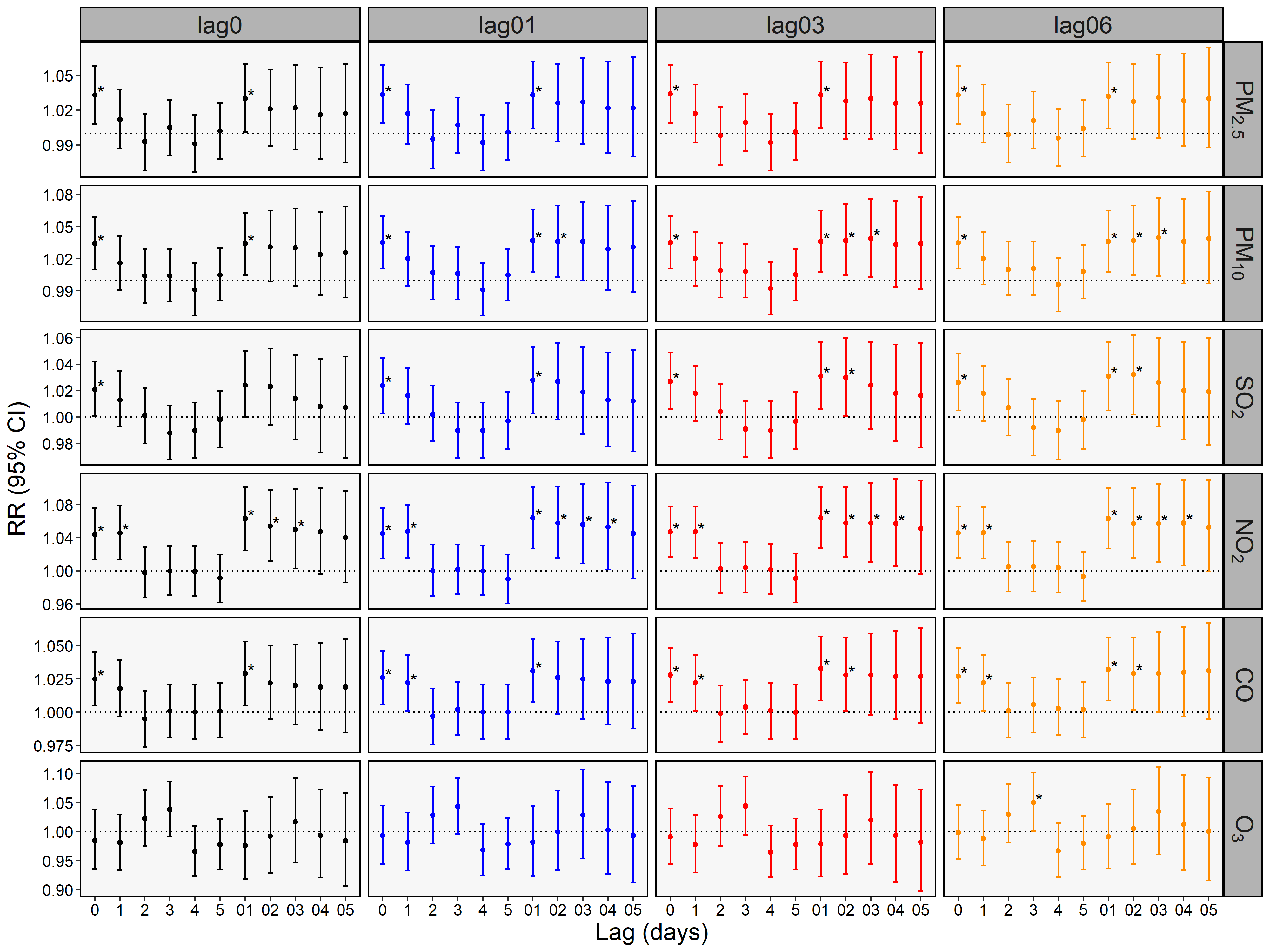


**Supplementary Figure 4.** Relative risks (RRs) with 95% CIs of in-hospital mortality for overall AMI per interquartile range (IQR) increase in air pollutant concentration adjusted the lag duration for temperature. Note: * indicates *P* <0.05. The IQR of daily concentrations of PM_2.5_, PM_10_, SO_2_, NO_2_, CO, and O_3_ were 63.33μg/m^3^, 74.23μg/m^3^, 10.30μg/m^3^, 26.91μg/m^3^, 0.67mg/m^3^ and 58.32μg/m^3^, respectively.

## Supplementary Tables

**Supplementary Table 1**. ICD-10 and ICD-9-CM codes of diagnosis and invasive procedure.

|  | **Code** |
| --- | --- |
| **Diagnosis** |  |
| Chronic obstructive pulmonary disease, COPD | J44 |
| Asthma | J45-J46 |
| Hypertension | I10-I15 |
| Diabetes | E10-E14 |
| Chronic kidney disease, CKD | N18-N19 |
| Stroke | I60-I64, I69 |
| Atrial flutter/atrial fibrillation, AFL/AF | I48 |
| Old myocardial infarction, OMI | I25.2 |
| Percutaneous coronary intervention history, PCI history | Z95.1, Z95.5 |
| Coronary artery bypass grafting history, CABG history | Z98.84 |
| Dyslipidemia | E78 |
| Heart failure | I50 |
| AMI mechanical complications | I23 |
| Ventricular tachycardia/ventricular fibrillation, VT/VF | I47.2, I49.0 |
| Pulmonary infection | J98.402 |
| **Invasive therapies and surgery** |  |
| Coronary artery bypass grafting, CABG | 36.10-36.17 |
| Percutaneous coronary intervention, PCI | 00.66, 17.55, 36.04001 |
| Coronary angiography, CAG | 88.55-88.57 |
| Intra-aortic balloon pump, IABP | 37.61003 |

ICD-10: International Classification of Disease, 10th Revision; ICD: International Classification of Diseases, 9th Revision, Clinical Modification.**Supplementary Table 2**. Demographic and clinical characteristics of all AMI population in Beijing during 2013-2019.

| **Variable** | **Overall AMI** | |  | **STEMI** | |  | **NSTEMI** | | ***P-*value^*^** |
| --- | --- | --- | --- | --- | --- | --- | --- | --- | --- |
|  | Deaths (n=10983) | Survival (n=138649) |  | Deaths (n=4361) | Survival  (n=66369) |  | Death  (n=4299) | Survival  (n=65295) |  |
| **Sex** |  |  |  |  |  |  |  |  | 0.316 |
| Male | 5942  (54.1) | 97764  (70.5) |  | 2379  (54.6) | 50491  (76.1) |  | 2299  (53.5) | 42703  (65.4) |  |
| Female | 5041  (45.9) | 40885  (29.5) |  | 1982  (45.4) | 15878  (23.9) |  | 2000  (46.5) | 22592  (34.6) |  |
| **Age** |  |  |  |  |  |  |  |  | 0.000 |
| <65 | 1367  (12.4) | 67864  (48.9) |  | 725  (16.6) | 38634  (58.2) |  | 382  (8.9) | 26511  (40.6) |  |
| ≥65 | 9616  (87.6) | 70785  (51.1) |  | 3636  (83.4) | 27735  (41.8) |  | 3917  (91.1) | 38784  (59.4) |  |
| **Comorbidities and complications** | | | | | | | | |  |
| Hypertension | 3525  (32.1) | 72162  (52.0) |  | 1482  (34.0) | 32752  (49.3) |  | 1356  (31.5) | 36038  (55.2) | 0.016 |
| Diabetes | 2203  (20.1) | 37157  (26.8) |  | 874  (20.0) | 16990  (25.6) |  | 875  (20.4) | 18370  (28.1) | 0.717 |
| Dyslipidemia | 1514  (13.8) | 61446  (44.3) |  | 697  (16.0) | 31837  (48.0) |  | 587  (13.7) | 27335  (41.9) | 0.002 |
| CKD | 1443  (13.1) | 7407  (5.3) |  | 460  (10.5) | 2308  (3.5) |  | 636  (14.8) | 4546  (7.0) | 0.000 |
| COPD | 373  (3.4) | 2302  (1.7) |  | 121  (2.8) | 779  (1.2) |  | 158  (3.7) | 1332  (2.0) | 0.018 |
| Asthma | 59  (0.5) | 911  (0.7) |  | 18  (0.4) | 395  (0.6) |  | 21  (0.5) | 461  (0.7) | 0.599 |
| Stroke | 1590  (14.5) | 13660  (9.9) |  | 589  (13.5) | 5426  (8.2) |  | 527  (12.3) | 7259  (11.1) | 0.083 |
| AFL/AF | 1991  (18.1) | 11034  (8.0) |  | 739  (16.9) | 3992  (6.0) |  | 903  (21.0) | 6417  (9.8) | 0.000 |
| OMI | 1525  (13.9) | 16180  (11.7) |  | 445  (10.2) | 4950  (7.5) |  | 840  (19.5) | 10445  (16.0) | 0.000 |
| PCI/CABG history | 908  (8.3) | 18351  (13.2) |  | 294  (6.7) | 7250  (10.9) |  | 465  (10.8) | 10232  (15.7) | 0.000 |
| AMI mechanical complications | 357  (3.3) | 165  (0.1) |  | 307  (7.0) | 140  (0.2) |  | 41  (1.0) | 16  (0.0) | 0.000 |
| Heart failure | 7316  (66.6) | 48243  (34.8) |  | 2841  (65.1) | 21093  (31.8) |  | 3126  (72.7) | 23873  (36.6) | 0.000 |
| VT/VF | 1257  (11.4) | 3458  (2.5) |  | 650  (14.9) | 2468  (3.7) |  | 467  (10.9) | 853  (1.3) | 0.000 |
| Pulmonary infection | 2758  (25.1) | 13058  (9.4) |  | 961  (22.0) | 4949  (7.5) |  | 1097  (25.5) | 6932  (10.6) | 0.000 |
| **Coma** | 38  (0.3) | 19  (0.0) |  | 19  (0.4) | 12  (0.0) |  | 10  (0.2) | 5  (0.0) | 0.102 |
| **Blood transfusion** | 808  (7.4) | 2663  (1.9) |  | 259  (5.9) | 933  (1.4) |  | 371  (8.6) | 1526  (2.3) | 0.000 |
| **Ventilator use** | 3892  (35.4) | 5209  (3.8) |  | 1730  (39.7) | 1970  (3.0) |  | 1449  (33.7) | 2736  (4.2) | 0.000 |
| **IABP use** | 628  (5.7) | 1754  (1.3) |  | 399  (9.1) | 1203  (1.8) |  | 174  (4.0) | 508  (0.8) | 0.000 |
| **Revascularization related operation/surgery** | | | | | | | | | 0.000 |
| CABG | 109  (1.0) | 2778  (2.0) |  | 34  (0.8) | 885  (1.3) |  | 58  (1.3) | 1668  (2.6) |  |
| PCI | 1209  (11.0) | 75253  (54.3) |  | 973  (22.3) | 46977  (70.8) |  | 165  (3.8) | 26122  (40.0) |  |
| only CAG | 382  (3.5) | 15600  (11.3) |  | 211  (4.8) | 5103  (7.7) |  | 132  (3.1) | 9836  (15.1) |  |
| No operation/surgery | 9283  (84.5) | 45018  (32.5) |  | 3143  (72.1) | 13404  (20.2) |  | 3944  (91.7) | 27669  (42.4) |  |
| **Medical payment type** | | | | | | | | | 0.000 |
| Basic medical insurance | 8137  (74.1) | 100667  (72.6) |  | 3155  (72.3) | 47015  (70.8) |  | 3306  (76.9) | 48995  (75.0) |  |
| Rural cooperative medical care | 881  (8.0) | 15123  (10.9) |  | 447  (10.2) | 7517  (11.3) |  | 257  (6.0) | 6662  (10.2) |  |
| Publicly funded medical care | 778  (7.1) | 6659  (4.8) |  | 252  (5.8) | 2909  (4.4) |  | 321  (7.5) | 3287  (5.0) |  |
| Self-pay | 497  (4.5) | 7219  (5.2) |  | 227  (5.2) | 4243  (6.4) |  | 169  (3.9) | 2606  (4.0) |  |
| Other | 690  (6.3) | 8981  (6.5) |  | 280  (6.4) | 4685  (7.1) |  | 246  (5.7) | 3745  (5.7) |  |
| **Marital status** | | | | | | | | | 0.000 |
| Unmarried | 276  (2.5) | 4789  (3.5) |  | 120  (2.8) | 2744  (4.1) |  | 90  (2.1) | 1898  (2.9) |  |
| Married | 9534  (86.8) | 127690  (92.1) |  | 3844  (88.1) | 61385  (92.5) |  | 3692  (85.9) | 59878  (91.7) |  |
| Widowed/divorced | 1173  (10.7) | 6170  (4.5) |  | 397  (9.1) | 2240  (3.4) |  | 517  (12.0) | 3519  (5.4) |  |
| **Occupation** |  |  |  |  |  |  |  |  | 0.000 |
| Employed | 2068  (18.8) | 41334  (29.8) |  | 989  (22.7) | 21625  (32.6) |  | 675  (15.7) | 17697  (27.1) |  |
| Unemployed | 588  (5.4) | 5878  (4.2) |  | 240  (5.5) | 2829  (4.3) |  | 207  (4.8) | 2558  (3.9) |  |
| Retired | 5804  (52.8) | 43934  (31.7) |  | 2010  (46.1) | 17663  (26.6) |  | 2435  (56.6) | 23405  (35.8) |  |
| Others | 2523  (23.0) | 47503  (34.3) |  | 1122  (25.7) | 24252  (36.5) |  | 982  (22.8) | 21635  (33.1) |  |

* *P*-value indicates between-group comparisons (STEMI vs. NSTEMI) in deceased patients, using chi-square test. *P*-values < 0.001 are shown as 0.000. COPD: chronic obstructive pulmonary disease; CKD, chronic kidney disease; AFL/AF, atrial flutter/atrial fibrillation; OMI, old myocardial infarction; CABG, coronary artery bypass grafting; PCI, percutaneous coronary intervention; VT/VF, ventricular tachycardia/ventricular fibrillation; CAG: coronary angiography; IABP, intra-aortic balloon pump.

**Supplementary Table 3**. Descriptive summary for meteorological factors.

|  | **Mean** | **SD** | **Minimum** | **P(25)** | **Median** | **P(75)** | **Maximum** |
| --- | --- | --- | --- | --- | --- | --- | --- |
| **Temperature** (℃) | 13.78 | 11.23 | -14.3 | 2.9 | 15.2 | 24.2 | 32.6 |
| **Relative humidity(%)** | 51.45 | 19.87 | 8 | 35 | 52 | 67 | 99 |
| **Air pressure(hPa)** | 1012.9 | 10.2 | 990 | 1004.2 | 1013 | 1021.1 | 1040 |

SD: standard deviation; P(25): 25th percentile; P(75): 75th percentile.

**Supplementary Table 4**. Spearman correlation analysis of air pollutants and meteorological factors.

|  | **PM_2.5_** | **PM_10_** | **SO_2_** | **NO_2_** | **CO** | **O_3_** | **Temperature** | **Relative humidity** |
| --- | --- | --- | --- | --- | --- | --- | --- | --- |
| **PM_10_** | 0.88* | - | - | - | - | - | - | - |
| **SO_2_** | 0.56* | 0.57* | - | - | - | - | - | - |
| **NO_2_** | 0.72* | 0.70* | 0.68* | - | - | - | - | - |
| **CO** | 0.87* | 0.71* | 0.65* | 0.76* | - | - | - | - |
| **O_3_** | -0.13* | -0.11* | -0.33* | -0.48* | -0.33* | - | - | - |
| **Temperature** | -0.04 | -0.03 | -0.46* | -0.31* | -0.21* | 0.72* | - | - |
| **Relative humidity** | 0.50* | 0.27* | -0.13* | 0.23* | 0.50* | -0.04* | 0.31* | - |
| **Air pressure** | -0.08* | -0.09* | 0.34* | 0.22* | 0.10* | -0.65* | -0.88* | -0.31* |

* indicates *P* <0.05.

**Supplementary Table 5**. Relative risks with 95% CIs of in-hospital mortality for overall AMI, STEMI, and NSTEMI per interquartile range (IQR) increase in air pollutant concentration: stratified by age.

|  | **Age** | **Overall AMI** | ***P_z_*** | **STEMI** | ***P_z_*** | **NSTEMI** | ***P_z_*** |
| --- | --- | --- | --- | --- | --- | --- | --- |
| **PM_2.5_** | <65 | 1.058(0.994,1.126) | 0.422 | 1.027(0.949,1.113) | 0.698 | 1.086(0.996,1.183) | 0.397 |
|  | ≥65 | 1.029(1.003,1.056)* |  | 1.009(0.968,1.052) |  | 1.042(1.002,1.084)* |  |
| **PM_10_** | <65 | 1.037(0.977,1.100) | 0.954 | 1.016(0.940,1.097) | 0.926 | 1.057(0.973,1.149) | 0.767 |
|  | ≥65 | 1.035(1.009,1.061)* |  | 1.020(0.978,1.064) |  | 1.043(1.004,1.083)* |  |
| **SO_2_** | <65 | 1.068(1.012,1.126)* | 0.126 | 0.979(0.913,1.050) | 0.320 | 1.040(0.963,1.123) | 0.847 |
|  | ≥65 | 1.020(0.997,1.044) |  | 1.019(0.982,1.057) |  | 1.049(1.012,1.087)* |  |
| **NO_2_** | <65 | 1.051(0.973,1.136) | 0.903 | 1.073(0.973,1.183) | 0.486 | 1.053(0.943,1.176) | 0.872 |
|  | ≥65 | 1.045(1.013,1.079)* |  | 1.031(0.980,1.085) |  | 1.063(1.013,1.116)* |  |
| **CO** | <65 | 1.046(0.992,1.102) | 0.483 | 1.033(0.967,1.105) | 0.518 | 1.050(0.975,1.131) | 0.808 |
|  | ≥65 | 1.025(1.003,1.047)* |  | 1.008(0.974,1.044) |  | 1.040(1.006,1.074)* |  |
| **O_3_** | <65 | 0.989(0.872,1.123) | 0.326 | 0.921(0.788,1.076) | 0.053 | 1.100(0.921,1.313) | 0.777 |
|  | ≥65 | 1.059(1.006,1.115)* |  | 1.095(1.010,1.187)* |  | 1.069(0.986,1.159) |  |

* indicates *P* <0.05; *P_z_*: *P* value for the between-group comparisons by Z test. The IQR of daily concentrations of PM_2.5_, PM_10_, SO_2_, NO_2_, CO, and O_3_ were 63.33μg/m^3^, 74.23μg/m^3^, 10.30μg/m^3^, 26.91μg/m^3^, 0.67mg/m^3^ and 58.32μg/m^3^, respectively. Based on the strongest effects in the single-day lag model, the lag days of PM_2.5_, PM_10_, NO_2_, and CO were selected as lag0 for overall AMI, STEMI, and NSTEMI. For SO_2_, the lag days were lag0 for overall AMI and NSTEMI, and lag5 for STEMI. For O_3_, lag days was lag3 for overall AMI, STEMI, and NSTEMI.

**Supplementary Table 6.** Relative risks with 95% CIs of in-hospital mortality for overall AMI, STEMI, and NSTEMI per interquartile range (IQR) increase in air pollutant concentration: stratified by sex.

|  | **Sex** | **Overall AMI** | ***P_z_*** | **STEMI** | ***P_z_*** | **NSTEMI** | ***P_z_*** |
| --- | --- | --- | --- | --- | --- | --- | --- |
| **PM_2.5_** | Male | 1.041(1.008,1.076)* | 0.471 | 1.010(0.958,1.064) | 0.918 | 1.054(1.001,1.110)* | 0.642 |
|  | Female | 1.023(0.989,1.059) |  | 1.014(0.961,1.070) |  | 1.036(0.984,1.091) |  |
| **PM_10_** | Male | 1.040(1.008,1.074)* | 0.596 | 1.017(0.965,1.072) | 0.956 | 1.040(0.990,1.091) | 0.830 |
|  | Female | 1.027(0.993,1.063) |  | 1.019(0.965,1.076) |  | 1.048(0.995,1.103) |  |
| **SO_2_** | Male | 1.039(1.010,1.069)* | 0.201 | 1.010(0.964,1.059) | 0.883 | 1.045(0.997,1.095) | 0.843 |
|  | Female | 1.011(0.981,1.042) |  | 1.015(0.969,1.064) |  | 1.052(1.004,1.102)* |  |
| **NO_2_** | Male | 1.062(1.020,1.105)* | 0.285 | 1.055(0.991,1.124) | 0.425 | 1.076(1.009,1.147)* | 0.583 |
|  | Female | 1.029(0.987,1.073) |  | 1.017(0.951,1.087) |  | 1.049(0.984,1.118) |  |
| **CO** | Male | 1.038(1.010,1.066)* | 0.288 | 1.014(0.970,1.059) | 0.910 | 1.053(1.008,1.101)* | 0.426 |
|  | Female | 1.016(0.988,1.045) |  | 1.010(0.966,1.056) |  | 1.028(0.985,1.072) |  |
| **O_3_** | Male | 1.095(1.026,1.168)* | 0.059 | 1.105(1.002,1.219)* | 0.266 | 1.100(0.990,1.223) | 0.499 |
|  | Female | 0.999(0.932,1.071) |  | 1.016(0.910,1.135) |  | 1.044(0.937,1.163) |  |

* indicates *P* <0.05; *P_z_*: *P* value for the between-group comparisons by Z test. The IQR of daily concentrations of PM_2.5_, PM_10_, SO_2_, NO_2_, CO, and O_3_ were 63.33μg/m^3^, 74.23μg/m^3^, 10.30μg/m^3^, 26.91μg/m^3^, 0.67mg/m^3^ and 58.32μg/m^3^, respectively. Based on the strongest effects in the single-day lag model, the lag days of PM_2.5_, PM_10_, NO_2_, and CO were selected as lag0 for overall AMI, STEMI, and NSTEMI. For SO_2_, the lag days were lag0 for overall AMI and NSTEMI, and lag5 for STEMI. For O_3_, lag days was lag3 for overall AMI, STEMI, and NSTEMI.

**Supplementary Table 7.** Relative risks with 95% CIs of in-hospital mortality for overall AMI, STEMI, and NSTEMI per interquartile range (IQR) increase in air pollutant concentration: stratified by hypertension.

|  | **Hypertension** | **Overall AMI** | ***P_z_*** | **STEMI** | ***P_z_*** | **NSTEMI** | ***P_z_*** |
| --- | --- | --- | --- | --- | --- | --- | --- |
| **PM_2.5_** | Yes | 1.045(1.005,1.087)* | 0.460 | 1.025(0.965,1.087) | 0.604 | 1.050(0.992,1.112) | 0.846 |
|  | No | 1.026(0.995,1.057) |  | 1.004(0.957,1.054) |  | 1.042(0.994,1.093) |  |
| **PM_10_** | Yes | 1.055(1.015,1.097)* | 0.232 | 1.045(0.982,1.112) | 0.310 | 1.053(0.996,1.113) | 0.709 |
|  | No | 1.024(0.995,1.054) |  | 1.004(0.957,1.053) |  | 1.038(0.992,1.087) |  |
| **SO_2_** | Yes | 1.026(0.992,1.062) | 0.996 | 1.015(0.965,1.069) | 0.904 | 1.070(1.016,1.126)* | 0.323 |
|  | No | 1.026(1.000,1.054) |  | 1.011(0.968,1.056) |  | 1.034(0.990,1.080) |  |
| **NO_2_** | Yes | 1.037(0.987,1.090) | 0.668 | 1.016(0.943,1.095) | 0.513 | 1.067(0.992,1.148) | 0.884 |
|  | No | 1.051(1.014,1.090)* |  | 1.049(0.990,1.111) |  | 1.060(1.001,1.122)* |  |
| **CO** | Yes | 1.031(0.998,1.065) | 0.797 | 1.010(0.962,1.060) | 0.923 | 1.053(1.003,1.106)* | 0.554 |
|  | No | 1.025(1.000,1.051)* |  | 1.013(0.973,1.055) |  | 1.033(0.994,1.074) |  |
| **O_3_** | Yes | 1.038(0.954,1.130) | 0.743 | 1.008(0.893,1.138) | 0.275 | 1.081(0.944,1.238) | 0.928 |
|  | No | 1.056(0.997,1.119) |  | 1.097(1.001,1.203)* |  | 1.073(0.980,1.175) |  |

* indicates *P* <0.05; *P_z_*: *P* value for the between-group comparisons by Z test. The IQR of daily concentrations of PM_2.5_, PM_10_, SO_2_, NO_2_, CO, and O_3_ were 63.33μg/m^3^, 74.23μg/m^3^, 10.30μg/m^3^, 26.91μg/m^3^, 0.67mg/m^3^ and 58.32μg/m^3^, respectively. Based on the strongest effects in the single-day lag model, the lag days of PM_2.5_, PM_10_, NO_2_, and CO were selected as lag0 for overall AMI, STEMI, and NSTEMI. For SO_2_, the lag days were lag0 for overall AMI and NSTEMI, and lag5 for STEMI. For O_3_, lag days was lag3 for overall AMI, STEMI, and NSTEMI.

**Supplementary Table 8.** Relative risks with 95% CIs of in-hospital mortality for overall AMI, STEMI, and NSTEMI per interquartile range (IQR) increase in air pollutant concentration: stratified by diabetes.

|  | **Diabetes** | **Overall AMI** | ***P_z_*** | **STEMI** | ***P_z_*** | **NSTEMI** | ***P_z_*** |
| --- | --- | --- | --- | --- | --- | --- | --- |
| **PM_2.5_** | Yes | 0.995(0.946,1.047) | 0.108 | 0.989(0.919,1.065) | 0.504 | 1.019(0.952,1.090) | 0.409 |
|  | No | 1.044(1.016,1.072)* |  | 1.018(0.976,1.063) |  | 1.054(1.009,1.100)* |  |
| **PM_10_** | Yes | 0.993(0.944,1.044) | 0.072 | 1.009(0.936,1.087) | 0.787 | 1.004(0.9400,1.073) | 0.211 |
|  | No | 1.046(1.019,1.074)* |  | 1.021(0.978,1.066) |  | 1.055(1.012,1.100)* |  |
| **SO_2_** | Yes | 1.027(0.982,1.074) | 0.959 | 1.018(0.958,1.083) | 0.836 | 1.101(1.035,1.172)* | 0.084 |
|  | No | 1.026(1.002,1.051)* |  | 1.011(0.972,1.050) |  | 1.032(0.993,1.073) |  |
| **NO_2_** | Yes | 0.983(0.923,1.047) | **0.031** | 0.950(0.867,1.041) | **0.041** | 1.024(0.941,1.115) | 0.363 |
|  | No | 1.063(1.029,1.099)* |  | 1.061(1.007,1.117)* |  | 1.073(1.017,1.132)* |  |
| **CO** | Yes | 0.987(0.947,1.029) | **0.032** | 0.948(0.892,1.009) | **0.023** | 1.023(0.969,1.080) | 0.503 |
|  | No | 1.039(1.016,1.063)* |  | 1.030(0.994,1.068) |  | 1.046(1.008,1.084)* |  |
| **O_3_** | Yes | 1.049(0.940,1.171) | 0.983 | 1.021(0.879,1.186) | 0.540 | 1.059(0.903,1.242) | 0.871 |
|  | No | 1.051(0.996,1.109) |  | 1.077(0.992,1.170) |  | 1.075(0.986,1.172) |  |

* indicates *P* <0.05; *P_z_*: *P* value for the between-group comparisons by Z test; bolding indicates *P_z_* <0.05. The IQR of daily concentrations of PM_2.5_, PM_10_, SO_2_, NO_2_, CO, and O_3_ were 63.33μg/m^3^, 74.23μg/m^3^, 10.30μg/m^3^, 26.91μg/m^3^, 0.67mg/m^3^ and 58.32μg/m^3^, respectively. Based on the strongest effects in the single-day lag model, the lag days of PM_2.5_, PM_10_, NO_2_, and CO were selected as lag0 for overall AMI, STEMI, and NSTEMI. For SO_2_, the lag days were lag0 for overall AMI and NSTEMI, and lag5 for STEMI. For O_3_, lag days was lag3 for overall AMI, STEMI, and NSTEMI.

**Supplementary Table 9.** Relative risks with 95% CIs of in-hospital mortality for overall AMI, STEMI, and NSTEMI per interquartile range (IQR) increase in air pollutant concentration: stratified by chronic kidney disease (CKD).

|  | **CKD** | **Overall AMI** | ***P_z_*** | **STEMI** | ***P_z_*** | **NSTEMI** | ***P_z_*** |
| --- | --- | --- | --- | --- | --- | --- | --- |
| **PM_2.5_** | Yes | 0.999(0.939,1.062) | 0.253 | 0.986(0.904,1.076) | 0.555 | 0.991(0.914,1.073) | 0.171 |
|  | No | 1.038(1.012,1.065)* |  | 1.015(0.976,1.056) |  | 1.055(1.013,1.099)* |  |
| **PM_10_** | Yes | 1.021(0.960,1.085) | 0.639 | 1.073(0.982,1.173) | 0.24 | 0.977(0.899,1.062) | 0.109 |
|  | No | 1.037(1.011,1.063)* |  | 1.012(0.973,1.054) |  | 1.053(1.013,1.095)* |  |
| **SO_2_** | Yes | 1.040(0.985,1.099) | 0.596 | 1.023(0.949,1.102) | 0.800 | 1.037(0.967,1.111) | 0.756 |
|  | No | 1.024(1.001,1.047)* |  | 1.012(0.977,1.048) |  | 1.050(1.012,1.089)* |  |
| **NO_2_** | Yes | 1.050(0.975,1.130） | 0.929 | 1.101(0.989,1.225) | 0.277 | 1.049(0.953,1.155) | 0.789 |
|  | No | 1.046(1.013,1.079)* |  | 1.031(0.983,1.082) |  | 1.065(1.013,1.120)* |  |
| **CO** | Yes | 0.999(0.951,1.050) | 0.249 | 0.995(0.924,1.071) | 0.636 | 1.008(0.946,1.074) | 0.311 |
|  | No | 1.032(1.010,1.054)* |  | 1.014(0.981,1.048) |  | 1.046(1.011,1.082)* |  |
| **O_3_** | Yes | 0.973(0.858,1.102) | 0.201 | 0.975(0.819,1.161) | 0.312 | 0.953(0.814,1.117) | 0.129 |
|  | No | 1.062(1.009,1.119)* |  | 1.076(0.996,1.162) |  | 1.096(1.007,1.192)* |  |

* indicates *P* <0.05; *P_z_*: *P* value for the between-group comparisons by Z test; CKD: chronic kidney disease. The IQR of daily concentrations of PM_2.5_, PM_10_, SO_2_, NO_2_, CO, and O_3_ were 63.33μg/m^3^, 74.23μg/m^3^, 10.30μg/m^3^, 26.91μg/m^3^, 0.67mg/m^3^ and 58.32μg/m^3^, respectively. Based on the strongest effects in the single-day lag model, the lag days of PM_2.5_, PM_10_, NO_2_, and CO were selected as lag0 for overall AMI, STEMI, and NSTEMI. For SO_2_, the lag days were lag0 for overall AMI and NSTEMI, and lag5 for STEMI. For O_3_, lag days was lag3 for overall AMI, STEMI, and NSTEMI.

**Supplementary Table 10.** Relative risks with 95% CIs of in-hospital mortality for overall AMI, STEMI, and NSTEMI per interquartile range (IQR) increase in air pollutant concentration: stratified by old myocardial infarction (OMI) history.

|  | **OMI**  **history** | **Overall AMI** | ***P_z_*** | **STEMI** | ***P_z_*** | **NSTEMI** | ***P_z_*** |
| --- | --- | --- | --- | --- | --- | --- | --- |
| **PM_2.5_** | Yes | 1.060(0.998,1.127) | 0.360 | 0.973(0.891,1.063) | 0.376 | 1.119(1.040,1.203)* | **0.048** |
|  | No | 1.028(1.002,1.055)* |  | 1.016(0.977,1.058) |  | 1.027(0.985,1.072) |  |
| **PM_10_** | Yes | 1.062(1.001,1.128)* | 0.354 | 0.988(0.905,1.080) | 0.503 | 1.086(1.011,1.167)* | 0.238 |
|  | No | 1.030(1.004,1.057)* |  | 1.022(0.982,1.063) |  | 1.033(0.992,1.076) |  |
| **SO_2_** | Yes | 1.010(0.958,1.065) | 0.521 | 1.024(0.949,1.104) | 0.774 | 1.054(0.985,1.128) | 0.853 |
|  | No | 1.029(1.006,1.053)* |  | 1.011(0.976,1.048) |  | 1.046(1.007,1.087)* |  |
| **NO_2_** | Yes | 1.065(0.989,1.148) | 0.604 | 1.043(0.938,1.160) | 0.918 | 1.096(1.000,1.200)* | 0.463 |
|  | No | 1.043(1.010,1.077)* |  | 1.037(0.987,1.088) |  | 1.054(1.000,1.110)* |  |
| **CO** | Yes | 1.044(0.993,1.097) | 0.497 | 0.940(0.871,1.015) | 0.057 | 1.115(1.050,1.185)* | **0.014** |
|  | No | 1.024(1.003,1.047)* |  | 1.020(0.986,1.054) |  | 1.022(0.987,1.059) |  |
| **O_3_** | Yes | 1.060(0.939,1.196) | 0.873 | 1.061(0.895,1.258) | 0.959 | 1.046(0.898,1.217) | 0.736 |
|  | No | 1.049(0.995,1.105) |  | 1.067(0.987,1.153) |  | 1.077(0.988,1.176) |  |

* indicates *P* <0.05; *P_z_*: *P* value for the between-group comparisons by Z test; bolding indicates *P_z_* <0.05; OMI: old myocardial infarction. The IQR of daily concentrations of PM_2.5_, PM_10_, SO_2_, NO_2_, CO, and O_3_ were 63.33μg/m^3^, 74.23μg/m^3^, 10.30μg/m^3^, 26.91μg/m^3^, 0.67mg/m^3^ and 58.32μg/m^3^, respectively. Based on the strongest effects in the single-day lag model, the lag days of PM_2.5_, PM_10_, NO_2_, and CO were selected as lag0 for overall AMI, STEMI, and NSTEMI. For SO_2_, the lag days were lag0 for overall AMI and NSTEMI, and lag5 for STEMI. For O_3_, lag days was lag3 for overall AMI, STEMI, and NSTEMI.

**Supplementary Table 11.** Relative risks with 95% CIs of in-hospital mortality for overall AMI, STEMI, and NSTEMI per interquartile range (IQR) increase in air pollutant concentration, stratified by PCI/CABG history

|  | **PCI/CABG history** | **Overall AMI** | ***P_z_*** | **STEMI** | ***P_z_*** | **NSTEMI** | ***P_z_*** |
| --- | --- | --- | --- | --- | --- | --- | --- |
| **PM_2.5_** | Yes | 1.059(0.983,1.141) | 0.507 | 0.970(0.879,1.071) | 0.408 | 1.106(1.012,1.209)* | 0.209 |
|  | No | 1.031(1.006,1.057)* |  | 1.015(0.976,1.056) |  | 1.040(1.000,1.081) |  |
| **PM_10_** | Yes | 1.047(0.979,1.120) | 0.724 | 0.965(0.889,1.048) | 0.210 | 1.092(1.006,1.185)* | 0.281 |
|  | No | 1.034(1.009,1.059)* |  | 1.023(0.983,1.065) |  | 1.039(1.000,1.079)* |  |
| **SO_2_** | Yes | 1.044(0.959,1.136) | 0.687 | 1.075(0.963,1.199) | 0.290 | 1.065(0.964,1.177) | 0.751 |
|  | No | 1.026(1.004,1.048)* |  | 1.010(0.976,1.046) |  | 1.047(1.011,1.084)* |  |
| **NO_2_** | Yes | 1.094(1.000,1.197) | 0.318 | 0.955(0.845,1.079) | 0.189 | 1.139(1.022,1.269)* | 0.206 |
|  | No | 1.042(1.011,1.075)* |  | 1.043(0.994,1.094) |  | 1.055(1.005,1.107)* |  |
| **CO** | Yes | 1.057(0.998,1.120) | 0.309 | 0.957(0.883,1.037) | 0.174 | 1.129(1.051,1.213)* | **0.024** |
|  | No | 1.024(1.004,1.046)* |  | 1.016(0.983,1.050) |  | 1.031(0.998,1.065) |  |
| **O_3_** | Yes | 1.067(0.926,1.230) | 0.830 | 0.972(0.812,1.164) | 0.332 | 1.146(0.973,1.350) | 0.416 |
|  | No | 1.049(0.998,1.103) |  | 1.071(0.991,1.157) |  | 1.062(0.979,1.153) |  |

* indicates *P* <0.05; *P_z_*: *P* value for the between-group comparisons by Z test; bolding indicates *P_z_* <0.05; PCI /CABG: percutaneous coronary intervention/coronary artery bypass grafting. The IQR of daily concentrations of PM_2.5_, PM_10_, SO_2_, NO_2_, CO, and O_3_ were 63.33μg/m^3^, 74.23μg/m^3^, 10.30μg/m^3^, 26.91μg/m^3^, 0.67mg/m^3^ and 58.32μg/m^3^, respectively. Based on the strongest effects in the single-day lag model, the lag days of PM_2.5_, PM_10_, NO_2_, and CO were selected as lag0 for overall AMI, STEMI, and NSTEMI. For SO_2_, the lag days were lag0 for overall AMI and NSTEMI, and lag5 for STEMI. For O_3_, lag days was lag3 for overall AMI, STEMI, and NSTEMI.

**Supplementary Table 12.** Odds ratios with 95% CIs of in-hospital mortality among overall AMI, STEMI, and NSTEMI patients per interquartile range (IQR) increase in air pollutant concentration in multivariable logistic regression in case-control analysis.

|  |  | **Overall AMI** | **STEMI** | **NSTEMI** |
| --- | --- | --- | --- | --- |
| **PM_2.5_** | **lag0** | 3.860(0.816,18.270) | 0.309(0.023,4.083) | 21.828(2.012,236.831)* |
|  | **lag1** | 1.057(0.218,5.129) | 0.183(0.013,2.534) | 5.173(0.467,57.345) |
|  | **lag2** | 1.151(0.236,5.625) | 0.172(0.012,2.406) | 2.244(0.195,25.826) |
|  | **lag3** | 2.277(0.478,10.845) | 0.691(0.051,9.362) | 2.985(0.269,33.119) |
|  | **lag4** | 1.543(0.314,7.577) | 1.069(0.080,14.353) | 0.559(0.046,6.849) |
|  | **lag5** | 2.758(0.565,13.478) | 0.347(0.026,4.722) | 1.116(0.091,13.731) |
|  | **lag01** | 2.457(0.423,14.280) | 0.167(0.009,3.137) | 19.170(1.309,280.849)* |
|  | **lag02** | 2.265(0.317,16.172) | 0.090(0.003,2.404) | 17.492(0.874,349.926) |
|  | **lag03** | 3.051(0.359,25.966) | 0.099(0.003,3.606) | 20.286(0.780,527.819) |
|  | **lag04** | 3.399(0.340,33.978) | 0.122(0.003,5.819) | 13.297(0.396,446.666) |
|  | **lag05** | 4.614(0.399,53.376) | 0.086(0.001,5.191) | 11.833(0.277,505.949) |
| **PM_10_** | **lag0** | 3.171(0.529,19.006) | 0.258(0.012,5.519) | 18.965(1.293,278.221)* |
|  | **lag1** | 0.696(0.111,4.361) | 0.146(0.007,3.045) | 5.789(0.367,91.344) |
|  | **lag2** | 1.951(0.311,12.231) | 0.763(0.034,17.183) | 2.251(0.138,36.772) |
|  | **lag3** | 1.752(0.276,11.111) | 1.108(0.052,23.570) | 1.704(0.097,29.784) |
|  | **lag4** | 0.700(0.106,4.612) | 0.376(0.017,8.469) | 0.720(0.040,13.059) |
|  | **lag5** | 1.036(0.166,6.451) | 0.179(0.008,3.830) | 1.273(0.075,21.569) |
|  | **lag01** | 1.695(0.217,13.256) | 0.123(0.004,3.903) | 20.460(0.932,449.210) |
|  | **lag02** | 2.201(0.222,21.861) | 0.152(0.003,7.238) | 18.773(0.588,599.241) |
|  | **lag03** | 2.611(0.214,31.859) | 0.199(0.003,13.408) | 17.056(0.387,752.531) |
|  | **lag04** | 2.108(0.144,30.939) | 0.151(0.002,13.814) | 12.239(0.209,717.598) |
|  | **lag05** | 2.001(0.116,34.398) | 0.082(0.001,9.835) | 11.299(0.150,850.181) |
| **SO_2_** | **lag0** | 1.180(0.993,1.403) | 0.899(0.677,1.192) | 1.421(1.086,1.858)* |
|  | **lag1** | 1.020(0.860,1.209) | 0.926(0.699,1.226) | 1.143(0.878,1.487) |
|  | **lag2** | 1.068(0.898,1.272) | 0.937(0.703,1.249) | 1.171(0.894,1.534) |
|  | **lag3** | 1.039(0.872,1.239) | 0.941(0.705,1.256) | 1.051(0.796,1.387) |
|  | **lag4** | 1.000(0.837,1.193) | 0.915(0.687,1.221) | 1.035(0.782,1.369) |
|  | **lag5** | 1.002(0.840,1.195) | 0.900(0.677,1.197) | 1.008(0.761,1.335) |
|  | **lag01** | 1.114(0.925,1.342) | 0.899(0.662,1.221) | 1.323(0.992,1.766) |
|  | **lag02** | 1.116(0.915,1.361) | 0.895(0.644,1.242) | 1.325(0.975,1.800) |
|  | **lag03** | 1.111(0.901,1.369) | 0.897(0.634,1.269) | 1.274(0.923,1.760) |
|  | **lag04** | 1.097(0.883,1.364) | 0.887(0.619,1.271) | 1.254(0.896,1.756) |
|  | **lag05** | 1.079(0.863,1.350) | 0.866(0.599,1.252) | 1.217(0.860,1.722) |
| **NO_2_** | **lag0** | 2.878(1.311,6.315)* | 1.295(0.352,4.755) | 4.645(1.388,15.546)* |
|  | **lag1** | 1.834(0.836,4.023) | 0.954(0.261,3.489) | 3.046(0.910,10.195) |
|  | **lag2** | 1.114(0.505,2.457) | 0.464(0.125,1.721) | 1.842(0.547,6.200) |
|  | **lag3** | 1.544(0.703,3.394) | 1.147(0.311,4.233) | 1.557(0.462,5.244) |
|  | **lag4** | 1.378(0.623,3.046) | 2.574(0.696,9.518) | 0.799(0.234,2.728) |
|  | **lag5** | 1.426(0.645,3.150) | 1.389(0.380,5.078) | 0.976(0.284,3.353) |
|  | **lag01** | 2.919(1.198,7.113)* | 1.158(0.265,5.065) | 5.438(1.384,21.362)* |
|  | **lag02** | 2.549(0.947,6.861) | 0.748(0.144,3.879) | 5.571(1.222,25.397)* |
|  | **lag03** | 2.800(0.956,8.200) | 0.847(0.141,5.081) | 5.397(1.043,27.943)* |
|  | **lag04** | 2.940(0.935,9.245) | 1.293(0.191,8.739) | 4.320(0.747,24.973) |
|  | **lag05** | 3.038(0.910,10.145) | 1.393(0.188,10.337) | 3.756(0.590,23.902) |
| **CO** | **lag0** | 1.018(1.005,1.031)* | 1.000(0.979,1.022) | 1.030(1.010,1.050)* |
|  | **lag1** | 1.007(0.994,1.020) | 0.996(0.975,1.018) | 1.015(0.995,1.035) |
|  | **lag2** | 1.005(0.992,1.018) | 0.990(0.968,1.012) | 1.010(0.990,1.030) |
|  | **lag3** | 1.011(0.998,1.024) | 1.000(0.978,1.022) | 1.012(0.992,1.032) |
|  | **lag4** | 1.012(0.998,1.025) | 1.009(0.988,1.032) | 1.007(0.987,1.028) |
|  | **lag5** | 1.013(0.999,1.026) | 0.999(0.977,1.021) | 1.006(0.986,1.028) |
|  | **lag01** | 1.016(1.001,1.030)* | 0.998(0.974,1.023) | 1.027(1.005,1.050)* |
|  | **lag02** | 1.015(0.999,1.031) | 0.993(0.967,1.020) | 1.027(1.003,1.052)* |
|  | **lag03** | 1.018(1.000,1.035)* | 0.995(0.966,1.024) | 1.028(1.002,1.054)* |
|  | **lag04** | 1.020(1.002,1.039)* | 0.999(0.969,1.030) | 1.028(1.000,1.057) |
|  | **lag05** | 1.023(1.003,1.042)* | 0.998(0.966,1.031) | 1.028(0.998,1.058) |
| **O_3_** | **lag0** | 0.358(0.042,3.060) | 0.233(0.007,7.802) | 0.757(0.028,20.691) |
|  | **lag1** | 1.582(0.185,13.522) | 1.355(0.041,44.831) | 4.662(0.169,128.960) |
|  | **lag2** | 4.262(0.500,36.297) | 12.084(0.359,406.441) | 7.968(0.293,216.507) |
|  | **lag3** | 3.995(0.458,34.825) | 5.737(0.176,186.581) | 7.553(0.262,217.609) |
|  | **lag4** | 1.286(0.146,11.364) | 0.455(0.013,15.634) | 1.564(0.052,46.937) |
|  | **lag5** | 1.962(0.227,16.926) | 1.367(0.040,46.925) | 5.391(0.198,146.668) |
|  | **lag01** | 0.709(0.070,7.126) | 0.505(0.012,22.031) | 2.082(0.059,73.297) |
|  | **lag02** | 1.456(0.129,16.435) | 1.759(0.033,93.961) | 4.132(0.099,172.991) |
|  | **lag03** | 2.167(0.174,27.025) | 2.895(0.047,178.831) | 6.311(0.129,309.671) |
|  | **lag04** | 2.088(0.154,28.271) | 1.958(0.028,137.602) | 5.556(0.099,313.112) |
|  | **lag05** | 2.250(0.156,32.374) | 1.906(0.024,149.238) | 6.908(0.113,422.889) |

* indicates *P* <0.05. The IQR of daily concentrations of PM_2.5_, PM_10_, SO_2_, NO_2_, CO, and O_3_ were 63.33μg/m^3^, 74.23μg/m^3^, 10.30μg/m^3^, 26.91μg/m^3^, 0.67mg/m^3^ and 58.32μg/m^3^, respectively.
